# Correction: Evidence-based conservation education in Mexican communities: Connecting arts and science

**DOI:** 10.1371/journal.pone.0244897

**Published:** 2020-12-29

**Authors:** The PLOS ONE Staff

The first author, Montserrat Franquesa-Soler, is incorrectly noted as the only corresponding author. The other corresponding author is Juan Carlos Serio-Silva. Juan Carlos Serio-Silva’s email address is: juan.serio@inecol.mx. The publisher apologizes for the error.
