# Supplementary material for: Evidence-based conservation education in Mexican communities: Connecting arts and science
Source: PLoS One. 2020 Feb 4;15(2):e0228382. doi: 10.1371/journal.pone.0228382 (PMC6999903; doi:10.1371/journal.pone.0228382)
Supplement: S1 Table — The dependent variables are the different factors considered during the implementation of the educational intervention (column 2–7) the independent variable is the Score (column 8). (DOCX) [file pone.0228382.s001.docx]

**Table 1.** Data set underlying the study of Evidence-based conservation education in Mexican communities: connecting arts and science. The dependent variables are the different factors considered during the implementation of the educational intervention (column 2-7) the independent variable is the Score (column 8). CONT: Context, urb: urban, rur: rural; PA: Protected Area; fem: Femenine, masc: masculine; SCH: school, pla: Playas del Rosario, jer: Jerusalén, ten: Redención del Campesino, ric: Puerto Rico, fra: San Francisco, cel: Celestún, cha: Champotón, xpu: Xpujil, bac: Bacalar, dzo: Dzoyolá, pal: ale: Punta Allen, pal: Nueva Palestina; TEC: technique used, thea: theater, sto: storytelling, sha: shadow puppets, con: control group; STA: State, tab: Tabasco, chi: Chiapas, cam: Campeche, yuc: Yucatan, qro: Quintana Roo; SCO: score.

| CODE | CONT | PA | SEX | SCH | TEC | STA | TIME | SCO |
| --- | --- | --- | --- | --- | --- | --- | --- | --- |
| UNF4Ab50 | urb | no | fem | pla | thea | tab | A | 3 |
| UNF1Ab51 | urb | no | fem | pla | sto | tab | A | 2 |
| UNM1Ab52 | urb | no | mas | pla | sto | tab | A | 2 |
| UNM3Ab53 | urb | no | mas | pla | sha | tab | A | 2 |
| UNF1Ab54 | urb | no | fem | pla | sto | tab | A | 2 |
| UNF3Ab55 | urb | no | fem | pla | sha | tab | A | 3 |
| UNM3Ab56 | urb | no | mas | pla | sha | tab | A | 3 |
| UNM1Ab57 | urb | no | mas | pla | sto | tab | A | 2 |
| UNF4Ab58 | urb | no | fem | pla | thea | tab | A | 2 |
| UNM3Ab59 | urb | no | mas | pla | sha | tab | A | 2 |
| UNM1Ab61 | urb | no | mas | pla | sto | tab | A | 1 |
| UNF2Ab62 | urb | no | fem | pla | con | tab | A | 2 |
| UNM4Ab65 | urb | no | mas | pla | thea | tab | A | 3 |
| UNF1Ab67 | urb | no | fem | pla | sto | tab | A | 3 |
| UNF2Ab70 | urb | no | fem | pla | con | tab | A | 2 |
| UNM3Ab71 | urb | no | mas | pla | sha | tab | A | 2 |
| UNF2Ab72 | urb | no | fem | pla | con | tab | A | 2 |
| UNM1Ab74 | urb | no | mas | pla | sto | tab | A | 2 |
| UNF1Ab75 | urb | no | fem | pla | sto | tab | A | 2 |
| UNM4Ab76 | urb | no | mas | pla | thea | tab | A | 1 |
| UNM4Ab79 | urb | no | mas | pla | thea | tab | A | 1 |
| UNF3Ab80 | urb | no | fem | pla | sha | tab | A | 2 |
| UNM4Ab81 | urb | no | mas | pla | thea | tab | A | 2 |
| UNF2Ab82 | urb | no | fem | pla | con | tab | A | 2 |
| UNM3Ab88 | urb | no | mas | pla | sha | tab | A | 1 |
| UNF3Ab89 | urb | no | fem | pla | sha | tab | A | 2 |
| UNF3Ab90 | urb | no | fem | pla | sha | tab | A | 2 |
| RNM2Ba01 | rur | no | mas | jer | con | chi | A | 2 |
| RNM3Ba02 | rur | no | mas | jer | sha | chi | A | 2 |
| RNM3Ba03 | rur | no | mas | jer | sha | chi | A | 2 |
| RNF1Ba04 | rur | no | fem | jer | sto | chi | A | 3 |
| RNF4Ba05 | rur | no | fem | jer | thea | chi | A | 3 |
| RNM2Ba06 | rur | no | mas | jer | con | chi | A | 4 |
| RNM2Ba07 | rur | no | mas | jer | con | chi | A | 2 |
| RNM3Ba08 | rur | no | mas | jer | sha | chi | A | 2 |
| RNF2Ba09 | rur | no | fem | jer | con | chi | A | 1 |
| RNF1Ba10 | rur | no | fem | jer | sto | chi | A | 3 |
| RNM3Ba11 | rur | no | mas | jer | sha | chi | A | 4 |
| RNM1Ba12 | rur | no | mas | jer | sto | chi | A | 2 |
| RNM4Ba14 | rur | no | mas | jer | thea | chi | A | 3 |
| RNM1Ba15 | rur | no | mas | jer | sto | chi | A | 5 |
| RNF2Ba16 | rur | no | fem | jer | con | chi | A | 3 |
| RNF2Ba17 | rur | no | fem | jer | con | chi | A | 2 |
| RNM3Ba18 | rur | no | mas | jer | sha | chi | A | 4 |
| RNM3Ba19 | rur | no | mas | jer | sha | chi | A | 2 |
| RNM1Ba20 | rur | no | mas | jer | sto | chi | A | 1 |
| RNF4Ba22 | rur | no | fem | jer | thea | chi | A | 2 |
| RNF1Ba23 | rur | no | fem | jer | sto | chi | A | 2 |
| RPM2Ca01 | rur | si | mas | ten | con | tab | A | 6 |
| RPF1Ca02 | rur | si | fem | ten | sto | tab | A | 1 |
| RPM2Ca03 | rur | si | mas | ten | con | tab | A | 3 |
| RPM1Ca04 | rur | si | mas | ten | sto | tab | A | 4 |
| RPF4Ca05 | rur | si | fem | ten | thea | tab | A | 3 |
| RPM3Ca06 | rur | si | mas | ten | sha | tab | A | 3 |
| RPM1Ca08 | rur | si | mas | ten | sto | tab | A | 4 |
| RPM4Ca09 | rur | si | mas | ten | thea | tab | A | 5 |
| RPM3Ca10 | rur | si | mas | ten | sha | tab | A | 4 |
| RPF2Ca11 | rur | si | fem | ten | con | tab | A | 5 |
| RPM1Ca13 | rur | si | mas | ten | sto | tab | A | 2 |
| RPM3Ca14 | rur | si | mas | ten | sha | tab | A | 4 |
| RPF3Ca16 | rur | si | fem | ten | sha | tab | A | 6 |
| RPM4Ca17 | rur | si | mas | ten | thea | tab | A | 4 |
| RPF2Ca18 | rur | si | fem | ten | con | tab | A | 4 |
| RPM3Ca20 | rur | si | mas | ten | sha | tab | A | 5 |
| RPM2Da01 | rur | si | mas | ric | con | cam | A | 4 |
| RPF3Da02 | rur | si | fem | ric | sha | cam | A | 3 |
| RPF3Da03 | rur | si | fem | ric | sha | cam | A | 4 |
| RPM1Da04 | rur | si | mas | ric | sto | cam | A | 6 |
| RPM4Da05 | rur | si | mas | ric | thea | cam | A | 3 |
| RPM2Da06 | rur | si | mas | ric | con | cam | A | 5 |
| RPF3Da07 | rur | si | fem | ric | sha | cam | A | 2 |
| RPF2Da08 | rur | si | fem | ric | con | cam | A | 3 |
| RPM2Da09 | rur | si | mas | ric | con | cam | A | 3 |
| RPF4Da10 | rur | si | fem | ric | thea | cam | A | 3 |
| RPM2Da11 | rur | si | mas | ric | con | cam | A | 6 |
| RPF2Da12 | rur | si | fem | ric | con | cam | A | 2 |
| RPM4Da14 | rur | si | mas | ric | thea | cam | A | 3 |
| RPF3Da15 | rur | si | fem | ric | sha | cam | A | 2 |
| RPM3Da16 | rur | si | mas | ric | sha | cam | A | 4 |
| RPM1Da19 | rur | si | mas | ric | sto | cam | A | 2 |
| RPF4Da22 | rur | si | fem | ric | thea | cam | A | 2 |
| RPM1Da23 | rur | si | mas | ric | sto | cam | A | 2 |
| RPF3Da24 | rur | si | fem | ric | sha | cam | A | 2 |
| RPF1Da25 | rur | si | fem | ric | sto | cam | A | 3 |
| RPF2Da26 | rur | si | fem | ric | con | cam | A | 2 |
| RPF5Da28 | rur | si | fem | ric | sha | cam | A | 2 |
| RPF4Da31 | rur | si | fem | ric | thea | cam | A | 2 |
| RPM3Da32 | rur | si | mas | ric | sha | cam | A | 3 |
| RPM1Da33 | rur | si | mas | ric | sto | cam | A | 3 |
| RNM4Ea01 | rur | no | mas | fra | thea | yuc | A | 2 |
| RNM2Ea02 | rur | no | mas | fra | con | yuc | A | 3 |
| RNF1Ea03 | rur | no | fem | fra | sto | yuc | A | 4 |
| RNM1Ea06 | rur | no | mas | fra | sto | yuc | A | 2 |
| RNM3Ea08 | rur | no | mas | fra | sha | yuc | A | 2 |
| RNF1Ea09 | rur | no | fem | fra | sto | yuc | A | 3 |
| RNF4Ea10 | rur | no | fem | fra | thea | yuc | A | 1 |
| RNM2Ea11 | rur | no | mas | fra | con | yuc | A | 2 |
| RNM4Ea12 | rur | no | mas | fra | thea | yuc | A | 6 |
| RNF1Ea13 | rur | no | fem | fra | sto | yuc | A | 3 |
| RNF1Ea14 | rur | no | fem | fra | sto | yuc | A | 2 |
| RNF2Ea16 | rur | no | fem | fra | con | yuc | A | 2 |
| RNM3Ea17 | rur | no | mas | fra | sha | yuc | A | 2 |
| RNF3Ea20 | rur | no | fem | fra | sha | yuc | A | 3 |
| RNM4Eb23 | rur | no | mas | fra | thea | yuc | A | 2 |
| RNF4Eb28 | rur | no | fem | fra | thea | yuc | A | 3 |
| RNF4Eb30 | rur | no | fem | fra | thea | yuc | A | 2 |
| RNF4Eb31 | rur | no | fem | fra | thea | yuc | A | 2 |
| RNF3Eb32 | rur | no | fem | fra | sha | yuc | A | 2 |
| RNM1Eb33 | rur | no | mas | fra | sto | yuc | A | 4 |
| RNF2Eb36 | rur | no | fem | fra | con | yuc | A | 3 |
| RNF1Eb39 | rur | no | fem | fra | sto | yuc | A | 3 |
| RNM1Eb42 | rur | no | mas | fra | sto | yuc | A | 2 |
| RNF1Eb43 | rur | no | fem | fra | sto | yuc | A | 4 |
| RNM2Eb44 | rur | no | mas | fra | con | yuc | A | 3 |
| UPM4Fa05 | urb | si | mas | cel | thea | yuc | A | 4 |
| UPF2Fa06 | urb | si | fem | cel | con | yuc | A | 4 |
| UPM1Fa07 | urb | si | mas | cel | sto | yuc | A | 3 |
| UPM3Fa08 | urb | si | mas | cel | sha | yuc | A | 4 |
| UPF3Fa09 | urb | si | fem | cel | sha | yuc | A | 1 |
| UPM4Fa10 | urb | si | mas | cel | thea | yuc | A | 3 |
| UPM4Fa14 | urb | si | mas | cel | thea | yuc | A | 3 |
| UPF1Fa15 | urb | si | fem | cel | sto | yuc | A | 4 |
| UPF3Fa16 | urb | si | fem | cel | sha | yuc | A | 3 |
| UPF2Fa19 | urb | si | fem | cel | con | yuc | A | 3 |
| UPF2Fa20 | urb | si | fem | cel | con | yuc | A | 3 |
| UPF2Fa21 | urb | si | fem | cel | con | yuc | A | 2 |
| UPM1Fa23 | urb | si | mas | cel | sto | yuc | A | 3 |
| UPM3Fa24 | urb | si | mas | cel | sha | yuc | A | 4 |
| UPM1Fa25 | urb | si | mas | cel | sto | yuc | A | 2 |
| UNM2Ga01 | urb | no | mas | cha | con | cam | A | 3 |
| UNM1Ga02 | urb | no | mas | cha | sto | cam | A | 3 |
| UNM2Ga03 | urb | no | mas | cha | con | cam | A | 2 |
| UNF3Ga04 | urb | no | fem | cha | sha | cam | A | 2 |
| UNF4Ga06 | urb | no | fem | cha | thea | cam | A | 2 |
| UNM2Ga07 | urb | no | mas | cha | con | cam | A | 5 |
| UNF1Ga08 | urb | no | fem | cha | sto | cam | A | 1 |
| UNF1Ga10 | urb | no | fem | cha | sto | cam | A | 2 |
| UNM3Ga11 | urb | no | mas | cha | sha | cam | A | 2 |
| UNM1Ga12 | urb | no | mas | cha | sto | cam | A | 3 |
| UNF4Ga13 | urb | no | fem | cha | thea | cam | A | 4 |
| UNF3Ga14 | urb | no | fem | cha | sha | cam | A | 3 |
| UNM2Ga15 | urb | no | mas | cha | con | cam | A | 3 |
| UNF3Ga16 | urb | no | fem | cha | sha | cam | A | 2 |
| UNF5Ga17 | urb | no | fem | cha | sha | cam | A | 2 |
| UNM4Ga18 | urb | no | mas | cha | thea | cam | A | 4 |
| UNM3Ga20 | urb | no | mas | cha | sha | cam | A | 3 |
| UNM4Ga23 | urb | no | mas | cha | thea | cam | A | 2 |
| UPF4Ha01 | urb | si | fem | xpu | thea | cam | A | 2 |
| UPF2Ha02 | urb | si | fem | xpu | con | cam | A | 2 |
| UPF1Ha03 | urb | si | fem | xpu | sto | cam | A | 2 |
| UPM3Ha06 | urb | si | mas | xpu | sha | cam | A | 5 |
| UPM1Ha07 | urb | si | mas | xpu | sto | cam | A | 2 |
| UPM2Ha08 | urb | si | mas | xpu | con | cam | A | 3 |
| UPF2Ha09 | urb | si | fem | xpu | con | cam | A | 2 |
| UPF1Ha10 | urb | si | fem | xpu | sto | cam | A | 4 |
| UPF1Ha12 | urb | si | fem | xpu | sto | cam | A | 2 |
| UPM3Ha13 | urb | si | mas | xpu | sha | cam | A | 2 |
| UPF4Ha16 | urb | si | fem | xpu | thea | cam | A | 3 |
| UPF2Ha17 | urb | si | fem | xpu | con | cam | A | 3 |
| UPM1Ha18 | urb | si | mas | xpu | sto | cam | A | 2 |
| UPF4Ha19 | urb | si | fem | xpu | thea | cam | A | 2 |
| UPM3Ha20 | urb | si | mas | xpu | sha | cam | A | 4 |
| UPF3Ha21 | urb | si | fem | xpu | sha | cam | A | 2 |
| UPF4Ha22 | urb | si | fem | xpu | thea | cam | A | 3 |
| UPF1Ha23 | urb | si | fem | xpu | sto | cam | A | 2 |
| UPF2Ha24 | urb | no | fem | xpu | con | cam | A | 1 |
| UNM2Ia05 | urb | no | mas | bac | con | qro | A | 1 |
| UNF4Ia07 | urb | no | fem | bac | thea | qro | A | 5 |
| UNM2Ia09 | urb | no | mas | bac | con | qro | A | 3 |
| UNF4Ia10 | urb | no | fem | bac | thea | qro | A | 4 |
| UNM4Ia11 | urb | no | mas | bac | thea | qro | A | 3 |
| UNF4Ia12 | urb | no | fem | bac | thea | qro | A | 2 |
| UNF1Ia13 | urb | no | fem | bac | sto | qro | A | 3 |
| UNM2Ia14 | urb | no | mas | bac | con | qro | A | 3 |
| UNF4Ia15 | urb | no | fem | bac | thea | qro | A | 2 |
| UNM2Ia16 | urb | no | mas | bac | con | qro | A | 3 |
| UNF1Ia17 | urb | no | fem | bac | sto | qro | A | 4 |
| UNF3Ia21 | urb | no | fem | bac | sha | qro | A | 2 |
| UNM4Ia24 | urb | no | mas | bac | thea | qro | A | 3 |
| UNM4Ia25 | urb | no | mas | bac | thea | qro | A | 4 |
| RNF1Ja02 | rur | no | fem | dzo | sto | qro | A | 2 |
| RNF3Ja03 | rur | no | fem | dzo | sha | qro | A | 3 |
| RNF4Ja04 | rur | no | fem | dzo | thea | qro | A | 4 |
| RNM2Ja05 | rur | no | mas | dzo | con | qro | A | 5 |
| RNF4Ja06 | rur | no | fem | dzo | thea | qro | A | 1 |
| RNF3Ja07 | rur | no | fem | dzo | sha | qro | A | 3 |
| RNF1Ja09 | rur | no | fem | dzo | sto | qro | A | 3 |
| RNM4Ja11 | rur | no | mas | dzo | thea | qro | A | 5 |
| RNF1Ja12 | rur | no | fem | dzo | sto | qro | A | 4 |
| RNF4Ja13 | rur | no | fem | dzo | thea | qro | A | 4 |
| RNF3Ja14 | rur | no | fem | dzo | sha | qro | A | 4 |
| RNM2Ja15 | rur | no | mas | dzo | con | qro | A | 4 |
| RNF2Ja16 | rur | no | fem | dzo | con | qro | A | 5 |
| RNM1Ja17 | rur | no | mas | dzo | sto | qro | A | 3 |
| RNF2Ja19 | rur | no | fem | dzo | con | qro | A | 3 |
| RNF1Ja20 | rur | no | fem | dzo | sto | qro | A | 3 |
| RNM2Ja21 | rur | no | mas | dzo | con | qro | A | 2 |
| RNF1Ja22 | rur | no | fem | dzo | sto | qro | A | 4 |
| RNF1Ja23 | rur | no | fem | dzo | sto | qro | A | 5 |
| RNM3Ja24 | rur | no | mas | dzo | sha | qro | A | 5 |
| RNF1Ja25 | rur | no | fem | dzo | sto | qro | A | 3 |
| RNF3Ja26 | rur | no | fem | dzo | sha | qro | A | 4 |
| RNF4Ja29 | rur | no | fem | dzo | thea | qro | A | 4 |
| UPF2La03 | urb | si | fem | pal | con | chi | A | 2 |
| UPF3La05 | urb | si | fem | pal | sha | chi | A | 3 |
| UPM2La07 | urb | si | mas | pal | con | chi | A | 3 |
| UPM1La10 | urb | si | mas | pal | sto | chi | A | 2 |
| UPF3La11 | urb | si | fem | pal | sha | chi | A | 2 |
| UPM1La14 | urb | si | mas | pal | sto | chi | A | 4 |
| UPM4La15 | urb | si | mas | pal | thea | chi | A | 4 |
| UPM3La16 | urb | si | mas | pal | sha | chi | A | 1 |
| UPM1La17 | urb | si | mas | pal | sto | chi | A | 5 |
| UPM3La18 | urb | si | mas | pal | sha | chi | A | 3 |
| UPM4La19 | urb | si | mas | pal | thea | chi | A | 3 |
| UPM1La20 | urb | si | mas | pal | sto | chi | A | 3 |
| UPM3La23 | urb | si | mas | pal | sha | chi | A | 4 |
| UPF4La24 | urb | si | fem | pal | thea | chi | A | 2 |
| UPM4La25 | urb | si | mas | pal | thea | chi | A | 3 |
| UPF1La26 | urb | si | fem | pal | sto | chi | A | 2 |
| UPM4La27 | urb | si | mas | pal | thea | chi | A | 3 |
| UPM2La30 | urb | si | mas | pal | con | chi | A | 2 |
| UPF3La31 | urb | si | fem | pal | sha | chi | A | 3 |
| RPF3Ka06 | rur | si | fem | ale | sha | qro | A | 5 |
| RPF3Ka12 | rur | si | fem | ale | sha | qro | A | 2 |
| RPM1Ka13 | rur | si | mas | ale | sto | qro | A | 2 |
| RPF2Ka19 | rur | si | fem | ale | con | qro | A | 3 |
| RPM1Ka26 | rur | si | mas | ale | sto | qro | A | 2 |
| RPF2Ka27 | rur | si | fem | ale | con | qro | A | 2 |
| RPF4Ka35 | rur | si | fem | ale | thea | qro | A | 2 |
| UNF4Ab50 | urb | no | fem | pla | thea | tab | **TIME** | 3 |
| UNF1Ab51 | urb | no | fem | pla | sto | tab | A | 4 |
| UNM1Ab52 | urb | no | mas | pla | sto | tab | A | 3 |
| UNM3Ab53 | urb | no | mas | pla | sha | tab | A | 3 |
| UNF1Ab54 | urb | no | fem | pla | sto | tab | A | 3 |
| UNF3Ab55 | urb | no | fem | pla | sha | tab | A | 4 |
| UNM3Ab56 | urb | no | mas | pla | sha | tab | A | 3 |
| UNM1Ab57 | urb | no | mas | pla | sto | tab | A | 1 |
| UNF4Ab58 | urb | no | fem | pla | thea | tab | A | 3 |
| UNM3Ab59 | urb | no | mas | pla | sha | tab | A | 3 |
| UNM1Ab61 | urb | no | mas | pla | sto | tab | A | 2 |
| UNF2Ab62 | urb | no | fem | pla | con | tab | A | 3 |
| UNM4Ab65 | urb | no | mas | pla | thea | tab | A | 2 |
| UNF1Ab67 | urb | no | fem | pla | sto | tab | A | 2 |
| UNF2Ab70 | urb | no | fem | pla | con | tab | A | 2 |
| UNM3Ab71 | urb | no | mas | pla | sha | tab | A | 5 |
| UNF2Ab72 | urb | no | fem | pla | con | tab | A | 3 |
| UNM1Ab74 | urb | no | mas | pla | sto | tab | A | 3 |
| UNF1Ab75 | urb | no | fem | pla | sto | tab | A | 3 |
| UNM4Ab76 | urb | no | mas | pla | thea | tab | A | 3 |
| UNM4Ab79 | urb | no | mas | pla | thea | tab | A | 3 |
| UNF3Ab80 | urb | no | fem | pla | sha | tab | A | 3 |
| UNM4Ab81 | urb | no | mas | pla | thea | tab | A | 3 |
| UNF2Ab82 | urb | no | fem | pla | con | tab | A | 2 |
| UNM3Ab88 | urb | no | mas | pla | sha | tab | A | 3 |
| UNF3Ab89 | urb | no | fem | pla | sha | tab | A | 2 |
| UNF3Ab90 | urb | no | fem | pla | sha | tab | A | 4 |
| RNM2Ba01 | rur | no | mas | jer | con | chi | A | 3 |
| RNM3Ba02 | rur | no | mas | jer | sha | chi | A | 4 |
| RNM3Ba03 | rur | no | mas | jer | sha | chi | A | 3 |
| RNF1Ba04 | rur | no | fem | jer | sto | chi | A | 4 |
| RNF4Ba05 | rur | no | fem | jer | thea | chi | A | 4 |
| RNM2Ba06 | rur | no | mas | jer | con | chi | A | 4 |
| RNM2Ba07 | rur | no | mas | jer | con | chi | A | 3 |
| RNM3Ba08 | rur | no | mas | jer | sha | chi | A | 1 |
| RNF2Ba09 | rur | no | fem | jer | con | chi | A | 5 |
| RNF1Ba10 | rur | no | fem | jer | sto | chi | A | 2 |
| RNM3Ba11 | rur | no | mas | jer | sha | chi | A | 2 |
| RNM1Ba12 | rur | no | mas | jer | sto | chi | A | 3 |
| RNM4Ba14 | rur | no | mas | jer | thea | chi | A | 1 |
| RNM1Ba15 | rur | no | mas | jer | sto | chi | A | 5 |
| RNF2Ba16 | rur | no | fem | jer | con | chi | A | 4 |
| RNF2Ba17 | rur | no | fem | jer | con | chi | A | 4 |
| RNM3Ba18 | rur | no | mas | jer | sha | chi | A | 5 |
| RNM3Ba19 | rur | no | mas | jer | sha | chi | A | 4 |
| RNM1Ba20 | rur | no | mas | jer | sto | chi | A | 3 |
| RNF4Ba22 | rur | no | fem | jer | thea | chi | A | 2 |
| RNF1Ba23 | rur | no | fem | jer | sto | chi | A | 2 |
| RPM2Ca01 | rur | si | mas | ten | con | tab | A | 5 |
| RPF1Ca02 | rur | si | fem | ten | sto | tab | A | 5 |
| RPM2Ca03 | rur | si | mas | ten | con | tab | A | 3 |
| RPM1Ca04 | rur | si | mas | ten | sto | tab | A | 4 |
| RPF4Ca05 | rur | si | fem | ten | thea | tab | A | 4 |
| RPM3Ca06 | rur | si | mas | ten | sha | tab | A | 3 |
| RPM1Ca08 | rur | si | mas | ten | sto | tab | A | 5 |
| RPM4Ca09 | rur | si | mas | ten | thea | tab | A | 6 |
| RPM3Ca10 | rur | si | mas | ten | sha | tab | A | 4 |
| RPF2Ca11 | rur | si | fem | ten | con | tab | A | 5 |
| RPM1Ca13 | rur | si | mas | ten | sto | tab | A | 3 |
| RPM3Ca14 | rur | si | mas | ten | sha | tab | A | 3 |
| RPF3Ca16 | rur | si | fem | ten | sha | tab | A | 6 |
| RPM4Ca17 | rur | si | mas | ten | thea | tab | A | 3 |
| RPF2Ca18 | rur | si | fem | ten | con | tab | A | 5 |
| RPM3Ca20 | rur | si | mas | ten | sha | tab | A | 5 |
| RPM2Da01 | rur | si | mas | ric | con | cam | A | 2 |
| RPF3Da02 | rur | si | fem | ric | sha | cam | A | 1 |
| RPF3Da03 | rur | si | fem | ric | sha | cam | A | 4 |
| RPM1Da04 | rur | si | mas | ric | sto | cam | A | 5 |
| RPM4Da05 | rur | si | mas | ric | thea | cam | A | 4 |
| RPM2Da06 | rur | si | mas | ric | con | cam | A | 3 |
| RPF3Da07 | rur | si | fem | ric | sha | cam | A | 2 |
| RPF2Da08 | rur | si | fem | ric | con | cam | A | 1 |
| RPM2Da09 | rur | si | mas | ric | con | cam | A | 2 |
| RPF4Da10 | rur | si | fem | ric | thea | cam | A | 6 |
| RPM2Da11 | rur | si | mas | ric | con | cam | A | 3 |
| RPF2Da12 | rur | si | fem | ric | con | cam | A | 3 |
| RPM4Da14 | rur | si | mas | ric | thea | cam | A | 4 |
| RPF3Da15 | rur | si | fem | ric | sha | cam | A | 1 |
| RPM3Da16 | rur | si | mas | ric | sha | cam | A | 2 |
| RPM1Da19 | rur | si | mas | ric | sto | cam | A | 4 |
| RPF4Da22 | rur | si | fem | ric | thea | cam | A | 4 |
| RPM1Da23 | rur | si | mas | ric | sto | cam | A | 3 |
| RPF3Da24 | rur | si | fem | ric | sha | cam | A | 4 |
| RPF1Da25 | rur | si | fem | ric | sto | cam | A | 4 |
| RPF2Da26 | rur | si | fem | ric | con | cam | A | 2 |
| RPF5Da28 | rur | si | fem | ric | sha | cam | A | 4 |
| RPF4Da31 | rur | si | fem | ric | thea | cam | A | 3 |
| RPM3Da32 | rur | si | mas | ric | sha | cam | A | 4 |
| RPM1Da33 | rur | si | mas | ric | sto | cam | A | 4 |
| RNM4Ea01 | rur | no | mas | fra | thea | yuc | A | 3 |
| RNM2Ea02 | rur | no | mas | fra | con | yuc | A | 3 |
| RNF1Ea03 | rur | no | fem | fra | sto | yuc | A | 3 |
| RNM1Ea06 | rur | no | mas | fra | sto | yuc | A | 3 |
| RNM3Ea08 | rur | no | mas | fra | sha | yuc | A | 2 |
| RNF1Ea09 | rur | no | fem | fra | sto | yuc | A | 1 |
| RNF4Ea10 | rur | no | fem | fra | thea | yuc | A | 3 |
| RNM2Ea11 | rur | no | mas | fra | con | yuc | A | 2 |
| RNM4Ea12 | rur | no | mas | fra | thea | yuc | A | 6 |
| RNF1Ea13 | rur | no | fem | fra | sto | yuc | A | 2 |
| RNF1Ea14 | rur | no | fem | fra | sto | yuc | A | 2 |
| RNF2Ea16 | rur | no | fem | fra | con | yuc | A | 3 |
| RNM3Ea17 | rur | no | mas | fra | sha | yuc | A | 3 |
| RNF3Ea20 | rur | no | fem | fra | sha | yuc | A | 5 |
| RNM4Eb23 | rur | no | mas | fra | thea | yuc | A | 3 |
| RNF4Eb28 | rur | no | fem | fra | thea | yuc | A | 4 |
| RNF4Eb30 | rur | no | fem | fra | thea | yuc | A | 4 |
| RNF4Eb31 | rur | no | fem | fra | thea | yuc | A | 4 |
| RNF3Eb32 | rur | no | fem | fra | sha | yuc | A | 3 |
| RNM1Eb33 | rur | no | mas | fra | sto | yuc | A | 3 |
| RNF2Eb36 | rur | no | fem | fra | con | yuc | A | 1 |
| RNF1Eb39 | rur | no | fem | fra | sto | yuc | A | 5 |
| RNM1Eb42 | rur | no | mas | fra | sto | yuc | A | 3 |
| RNF1Eb43 | rur | no | fem | fra | sto | yuc | A | 5 |
| RNM2Eb44 | rur | no | mas | fra | con | yuc | A | 4 |
| UPM4Fa05 | urb | si | mas | cel | thea | yuc | A | 4 |
| UPF2Fa06 | urb | si | fem | cel | con | yuc | A | 4 |
| UPM1Fa07 | urb | si | mas | cel | sto | yuc | A | 4 |
| UPM3Fa08 | urb | si | mas | cel | sha | yuc | A | 4 |
| UPF3Fa09 | urb | si | fem | cel | sha | yuc | A | 2 |
| UPM4Fa10 | urb | si | mas | cel | thea | yuc | A | 3 |
| UPM4Fa14 | urb | si | mas | cel | thea | yuc | A | 4 |
| UPF1Fa15 | urb | si | fem | cel | sto | yuc | A | 3 |
| UPF3Fa16 | urb | si | fem | cel | sha | yuc | A | 3 |
| UPF2Fa19 | urb | si | fem | cel | con | yuc | A | 2 |
| UPF2Fa20 | urb | si | fem | cel | con | yuc | A | 4 |
| UPF2Fa21 | urb | si | fem | cel | con | yuc | A | 4 |
| UPM1Fa23 | urb | si | mas | cel | sto | yuc | A | 5 |
| UPM3Fa24 | urb | si | mas | cel | sha | yuc | A | 4 |
| UPM1Fa25 | urb | si | mas | cel | sto | yuc | A | 5 |
| UNM2Ga01 | urb | no | mas | cha | con | cam | A | 5 |
| UNM1Ga02 | urb | no | mas | cha | sto | cam | A | 5 |
| UNM2Ga03 | urb | no | mas | cha | con | cam | A | 4 |
| UNF3Ga04 | urb | no | fem | cha | sha | cam | A | 4 |
| UNF4Ga06 | urb | no | fem | cha | thea | cam | A | 4 |
| UNM2Ga07 | urb | no | mas | cha | con | cam | A | 4 |
| UNF1Ga08 | urb | no | fem | cha | sto | cam | A | 5 |
| UNF1Ga10 | urb | no | fem | cha | sto | cam | A | 6 |
| UNM3Ga11 | urb | no | mas | cha | sha | cam | A | 5 |
| UNM1Ga12 | urb | no | mas | cha | sto | cam | A | 4 |
| UNF4Ga13 | urb | no | fem | cha | thea | cam | A | 5 |
| UNF3Ga14 | urb | no | fem | cha | sha | cam | A | 4 |
| UNM2Ga15 | urb | no | mas | cha | con | cam | A | 3 |
| UNF3Ga16 | urb | no | fem | cha | sha | cam | A | 5 |
| UNF5Ga17 | urb | no | fem | cha | sha | cam | A | 5 |
| UNM4Ga18 | urb | no | mas | cha | thea | cam | A | 6 |
| UNM3Ga20 | urb | no | mas | cha | sha | cam | A | 5 |
| UNM4Ga23 | urb | no | mas | cha | thea | cam | A | 3 |
| UPF4Ha01 | urb | si | fem | xpu | thea | cam | A | 4 |
| UPF2Ha02 | urb | si | fem | xpu | con | cam | A | 2 |
| UPF1Ha03 | urb | si | fem | xpu | sto | cam | A | 3 |
| UPM3Ha06 | urb | si | mas | xpu | sha | cam | A | 5 |
| UPM1Ha07 | urb | si | mas | xpu | sto | cam | A | 5 |
| UPM2Ha08 | urb | si | mas | xpu | con | cam | A | 5 |
| UPF2Ha09 | urb | si | fem | xpu | con | cam | A | 2 |
| UPF1Ha10 | urb | si | fem | xpu | sto | cam | A | 4 |
| UPF1Ha12 | urb | si | fem | xpu | sto | cam | A | 2 |
| UPM3Ha13 | urb | si | mas | xpu | sha | cam | A | 4 |
| UPF4Ha16 | urb | si | fem | xpu | thea | cam | A | 5 |
| UPF2Ha17 | urb | si | fem | xpu | con | cam | A | 4 |
| UPM1Ha18 | urb | si | mas | xpu | sto | cam | A | 5 |
| UPF4Ha19 | urb | si | fem | xpu | thea | cam | A | 2 |
| UPM3Ha20 | urb | si | mas | xpu | sha | cam | A | 3 |
| UPF3Ha21 | urb | si | fem | xpu | sha | cam | A | 2 |
| UPF4Ha22 | urb | si | fem | xpu | thea | cam | A | 4 |
| UPF1Ha23 | urb | si | fem | xpu | sto | cam | A | 4 |
| UPF2Ha24 | urb | no | fem | xpu | con | cam | A | 2 |
| UNM2Ia05 | urb | no | mas | bac | con | qro | A | 3 |
| UNF4Ia07 | urb | no | fem | bac | thea | qro | A | 5 |
| UNM2Ia09 | urb | no | mas | bac | con | qro | A | 5 |
| UNF4Ia10 | urb | no | fem | bac | thea | qro | A | 4 |
| UNM4Ia11 | urb | no | mas | bac | thea | qro | A | 3 |
| UNF4Ia12 | urb | no | fem | bac | thea | qro | A | 3 |
| UNF1Ia13 | urb | no | fem | bac | sto | qro | A | 5 |
| UNM2Ia14 | urb | no | mas | bac | con | qro | A | 5 |
| UNF4Ia15 | urb | no | fem | bac | thea | qro | A | 2 |
| UNM2Ia16 | urb | no | mas | bac | con | qro | A | 2 |
| UNF1Ia17 | urb | no | fem | bac | sto | qro | A | 6 |
| UNF3Ia21 | urb | no | fem | bac | sha | qro | A | 2 |
| UNM4Ia24 | urb | no | mas | bac | thea | qro | A | 3 |
| UNM4Ia25 | urb | no | mas | bac | thea | qro | A | 4 |
| RNF1Ja02 | rur | no | fem | dzo | sto | qro | A | 5 |
| RNF3Ja03 | rur | no | fem | dzo | sha | qro | A | 2 |
| RNF4Ja04 | rur | no | fem | dzo | thea | qro | A | 4 |
| RNM2Ja05 | rur | no | mas | dzo | con | qro | A | 5 |
| RNF4Ja06 | rur | no | fem | dzo | thea | qro | A | 3 |
| RNF3Ja07 | rur | no | fem | dzo | sha | qro | A | 3 |
| RNF1Ja09 | rur | no | fem | dzo | sto | qro | A | 6 |
| RNM4Ja11 | rur | no | mas | dzo | thea | qro | A | 4 |
| RNF1Ja12 | rur | no | fem | dzo | sto | qro | A | 4 |
| RNF4Ja13 | rur | no | fem | dzo | thea | qro | A | 5 |
| RNF3Ja14 | rur | no | fem | dzo | sha | qro | A | 5 |
| RNM2Ja15 | rur | no | mas | dzo | con | qro | A | 4 |
| RNF2Ja16 | rur | no | fem | dzo | con | qro | A | 2 |
| RNM1Ja17 | rur | no | mas | dzo | sto | qro | A | 5 |
| RNF2Ja19 | rur | no | fem | dzo | con | qro | A | 4 |
| RNF1Ja20 | rur | no | fem | dzo | sto | qro | A | 6 |
| RNM2Ja21 | rur | no | mas | dzo | con | qro | A | 5 |
| RNF1Ja22 | rur | no | fem | dzo | sto | qro | A | 6 |
| RNF1Ja23 | rur | no | fem | dzo | sto | qro | A | 5 |
| RNM3Ja24 | rur | no | mas | dzo | sha | qro | A | 4 |
| RNF1Ja25 | rur | no | fem | dzo | sto | qro | A | 6 |
| RNF3Ja26 | rur | no | fem | dzo | sha | qro | A | 5 |
| RNF4Ja29 | rur | no | fem | dzo | thea | qro | A | 6 |
| UPF2La03 | urb | si | fem | pal | con | chi | A | 3 |
| UPF3La05 | urb | si | fem | pal | sha | chi | A | 4 |
| UPM2La07 | urb | si | mas | pal | con | chi | A | 1 |
| UPM1La10 | urb | si | mas | pal | sto | chi | A | 5 |
| UPF3La11 | urb | si | fem | pal | sha | chi | A | 4 |
| UPM1La14 | urb | si | mas | pal | sto | chi | A | 5 |
| UPM4La15 | urb | si | mas | pal | thea | chi | A | 5 |
| UPM3La16 | urb | si | mas | pal | sha | chi | A | 3 |
| UPM1La17 | urb | si | mas | pal | sto | chi | A | 4 |
| UPM3La18 | urb | si | mas | pal | sha | chi | A | 6 |
| UPM4La19 | urb | si | mas | pal | thea | chi | A | 6 |
| UPM1La20 | urb | si | mas | pal | sto | chi | A | 4 |
| UPM3La23 | urb | si | mas | pal | sha | chi | A | 5 |
| UPF4La24 | urb | si | fem | pal | thea | chi | A | 4 |
| UPM4La25 | urb | si | mas | pal | thea | chi | A | 5 |
| UPF1La26 | urb | si | fem | pal | sto | chi | A | 3 |
| UPM4La27 | urb | si | mas | pal | thea | chi | A | 4 |
| UPM2La30 | urb | si | mas | pal | con | chi | A | 2 |
| UPF3La31 | urb | si | fem | pal | sha | chi | A | 6 |
| RPF3Ka06 | rur | si | fem | ale | sha | qro | A | 3 |
| RPF3Ka12 | rur | si | fem | ale | sha | qro | A | 4 |
| RPM1Ka13 | rur | si | mas | ale | sto | qro | A | 4 |
| RPF2Ka19 | rur | si | fem | ale | con | qro | A | 2 |
| RPM1Ka26 | rur | si | mas | ale | sto | qro | A | 3 |
| RPF2Ka27 | rur | si | fem | ale | con | qro | A | 2 |
| RPF4Ka35 | rur | si | fem | ale | thea | qro | A | 6 |
