# Supplementary material for: Evidence-based conservation education in Mexican communities: Connecting arts and science
Source: PLoS One. 2020 Feb 4;15(2):e0228382. doi: 10.1371/journal.pone.0228382 (PMC6999903; doi:10.1371/journal.pone.0228382)
Supplement: Attachment — Submitted filename: PONE-D-19-15156_reviewer (1) Art and education Mexico.pdf [file pone.0228382.s002.pdf]

PLOS ONE

Evidence-based conservation education in Mexican communities: connecting arts and science

--Manuscript Draft--

**Manuscript Number:**	PONE-D-19-15156
**Article Type:**	Research Article
**Full Title:**	Evidence-based conservation education in Mexican communities: connecting arts and science
**Short Title:**	Conservation through arts-and-science-based education in Mexico
**Corresponding Author:**	Montserrat Franquesa-Soler<br>Universidad Nacional Autonoma de Mexico Instituto de Investigaciones en Ecosistemas y Sustentabilidad<br>Morelia, MEXICO
**Keywords:**	Arts for education; Conservation Education; Drawings analysis; Holistic experience; Mexico; Primates
**Abstract:**	Several studies suggest that 63% of primate spp. are currently threatened due to deforestation, pet-trade, and bushmeat hunting. Successful primate conservation strategies require accurate educational programs capable of enhancing new system-thinking and responsible behavior with these species. Arts-based conservation education can inclusively foster cognitive and emotional processes. In this paper, we evaluate an arts-based Primate Conservation Education program conducted in Southern Mexico. A total of 229 children from habitat communities participated in a program for the conservation of black howler monkeys (Alouatta pigra). Different teaching methods were tested (storytelling, theater and shadow puppets), contrasted with a control group, and evaluated through a drawings analysis. Our results showed that children's knowledge score was predicted by the technique used and the residence within or outside of Protected Areas (PAs). Conversely, gender and context (urban or rural) did not. Overall, indicators revealed an increase of knowledge and a decrease of misconceptions between Pre-Post evaluations. Finally, a satisfaction survey about the program showed a high positive feedback. The study highlights the value of designing multidisciplinary projects based on holistic experiences, where the arts-based education program (grounded in previous scientific studies) has shown to be a successful way to conduct a Primate Conservation Education program.
**Order of Authors:**	Montserrat Franquesa-Soler
	Lucía Jorge-Sales
	Patricia Moreno-Casasola
	Juan Carlos Serio-Silva
**Additional Information:**	
**Question**	**Response**
**Financial Disclosure**<br><br>Enter a financial disclosure statement that describes the sources of funding for the work included in this submission. Review the submission guidelines for detailed requirements. View published research articles from *PLOS ONE* for specific examples.<br><br>This statement is required for submission and **will appear in the published article** if	We thank Consejo Nacional de Ciencia y Tecnología (CONACyT, Mexico) for offering the scholarship to MFS (N° 556384), Instituto de Ecología, A.C (INECOL, A.C, Mexico), DGAPA-UNAM Postdoctoral Fellowship, and to JCSS for financial support.

Powered by Editorial Manager® and ProduXion Manager® from Aries Systems Corporation

the submission is accepted. Please make sure it is accurate.<br><br>**Unfunded studies**<br>Enter: *The author(s) received no specific funding for this work.*<br><br>**Funded studies**<br>Enter a statement with the following details:<br>• Initials of the authors who received each award<br>• Grant numbers awarded to each author<br>• The full name of each funder<br>• URL of each funder website<br>• Did the sponsors or funders play any role in the study design, data collection and analysis, decision to publish, or preparation of the manuscript?<br>• **NO** - Include this sentence at the end of your statement: *The funders had no role in study design, data collection and analysis, decision to publish, or preparation of the manuscript.*<br>• **YES** - Specify the role(s) played.<br><br>* typeset	
**Competing Interests**<br><br>Use the instructions below to enter a competing interest statement for this submission. On behalf of all authors, disclose any competing interests that could be perceived to bias this work—acknowledging all financial support and any other relevant financial or non-financial competing interests.<br><br>This statement **will appear in the published article** if the submission is accepted. Please make sure it is accurate. View published research articles from *PLOS ONE* for specific examples.	The authors have declared that no competing interests exist.

**NO authors have competing interests**<br><br>Enter: *The authors have declared that no competing interests exist.*<br><br>**Authors with competing interests**<br><br>Enter competing interest details beginning with this statement:<br><br>*I have read the journal's policy and the authors of this manuscript have the following competing interests: [insert competing interests here]*<br><br>* typeset	
**Ethics Statement**<br><br>Enter an ethics statement for this submission. This statement is required if the study involved:<br><br>• Human participants<br>• Human specimens or tissue<br>• Vertebrate animals or cephalopods<br>• Vertebrate embryos or tissues<br>• Field research<br><br>Write "N/A" if the submission does not require an ethics statement.<br><br>General guidance is provided below. Consult the submission guidelines for detailed instructions. **Make sure that all information entered here is included in the Methods section of the manuscript.**	All primary schools agreed to join our educational intervention and official permissions were obtained by Institutional review boards of School Council and Secretaria de Educación Pública - Secretariat of Public Education- in the Governement office of each state (Documents attached as supplementary material)

Powered by Editorial Manager® and ProduXion Manager® from Aries Systems Corporation

**Format for specific study types**<br><br>**Human Subject Research (involving human participants and/or tissue)**<br>• Give the name of the institutional review board or ethics committee that approved the study<br>• Include the approval number and/or a statement indicating approval of this research<br>• Indicate the form of consent obtained (written/oral) or the reason that consent was not obtained (e.g. the data were analyzed anonymously)<br><br>**Animal Research (involving vertebrate animals, embryos or tissues)**<br>• Provide the name of the Institutional Animal Care and Use Committee (IACUC) or other relevant ethics board that reviewed the study protocol, and indicate whether they approved this research or granted a formal waiver of ethical approval<br>• Include an approval number if one was obtained<br>• If the study involved *non-human primates*, add *additional details* about animal welfare and steps taken to ameliorate suffering<br>• If anesthesia, euthanasia, or any kind of animal sacrifice is part of the study, include briefly which substances and/or methods were applied<br><br>**Field Research**<br><br>Include the following details if this study involves the collection of plant, animal, or other materials from a natural setting:<br>• Field permit number<br>• Name of the institution or relevant body that granted permission	
**Data Availability**<br><br>Authors are required to make all data underlying the findings described fully available, without restriction, and from the time of publication. PLOS allows rare exceptions to address legal and ethical concerns. See the PLOS Data Policy and FAQ for detailed information.	Yes - all data are fully available without restriction; Yes - all data are fully available without restriction

A Data Availability Statement describing where the data can be found is required at submission. Your answers to this question constitute the Data Availability Statement and **will be published in the article**, if accepted.<br><br>**Important:** Stating 'data available on request from the author' is not sufficient. If your data are only available upon request, select 'No' for the first question and explain your exceptional situation in the text box.<br><br>Do the authors confirm that all data underlying the findings described in their manuscript are fully available without restriction?	
**Describe where the data may be found in full sentences. If you are copying our sample text, replace any instances of XXX with the appropriate details.**<br><br>• If the data are **held or will be held in a public repository**, include URLs, accession numbers or DOIs. If this information will only be available after acceptance, indicate this by ticking the box below. For example: *All XXX files are available from the XXX database (accession number(s) XXX, XXX.*).<br>• If the data are all contained **within the manuscript and/or Supporting Information files**, enter the following: *All relevant data are within the manuscript and its Supporting Information files.*<br>• If neither of these applies but you are able to provide **details of access elsewhere**, with or without limitations, please do so. For example:<br><br>*Data cannot be shared publicly because of [XXX]. Data are available from the XXX Institutional Data Access / Ethics Committee (contact via XXX) for researchers who meet the criteria for access to confidential data.*<br><br>*The data underlying the results presented in the study are available from (include the name of the third party*	N/AN/A

and contact information or URL).<br>• This text is appropriate if the data are owned by a third party and authors do not have permission to share the data.<br><br>* typeset	
Additional data availability information:	Tick here if the URLs/accession numbers/DOIs will be available only after acceptance of the manuscript for publication so that we can ensure their inclusion before publication.; Tick here if the URLs/accession numbers/DOIs will be available only after acceptance of the manuscript for publication so that we can ensure their inclusion before publication.

Powered by Editorial Manager® and ProduXion Manager® from Aries Systems Corporation

Manuscript Click here to download Manuscript Manuscript.docx

Evidence-based conservation education in Mexican communities: connecting arts and science

Short title: Conservation through arts-and-science-based education in Mexico

Franquesa-Soler, M. [1,2,#a], Jorge-Sales[2], L., Moreno-Casasola, P[3,] Serio-Silva, J.C[4]

[1] Posgrado, Instituto de Ecología AC, Xalapa, Veracruz, México

[2] Department of Primate Conservation and Sustainable Development, Miku Conservación AC, Xalapa, Veracruz, México.

[3] Red de Ecología Funcional, Instituto de Ecología AC, Xalapa, Veracruz, México.

[4] Red de Biología y Conservación de Vertebrados, Instituto de Ecología AC, Xalapa, Veracruz, México.

[#a]Current address: Laboratorio de Interacciones Planta-Animal, Instituto de Investigaciones en Ecosistemas y Sustentabilidad, Universidad Autónoma de México (IIES-UNAM), Morelia, Michoacán, México.

Corresponding authors *

E-mail: franquesamontse@gmail.com (MFS)

E-mail: juan.serio@inecol.mx (JSS)

Abstract

Several studies suggest that 63% of primate spp. are currently threatened due to deforestation, pet-trade, and bushmeat hunting. Successful primate conservation strategies require accurate educational programs capable of enhancing new system-thinking and responsible behavior with these species. Arts-based conservation education can inclusively foster cognitive and emotional processes. In this paper, we evaluate an arts-based Primate Conservation Education program conducted in Southern Mexico. A total of 229 children from habitat communities participated in a program for the conservation of black howler monkeys (*Alouatta pigra*). Different teaching methods were tested (storytelling, theater and shadow puppets), contrasted with a control group, and evaluated through a drawings analysis. Our results showed that children's knowledge score was predicted by the technique used and the residence within or outside of Protected Areas (PAs). Conversely, gender and context (urban or rural) did not. Overall, indicators revealed an increase of knowledge and a decrease of misconceptions between Pre-Post evaluations. Finally, a satisfaction survey about the program showed a high positive feedback. The study highlights the value of designing multidisciplinary projects based on holistic experiences, where the arts-based education program (grounded in previous scientific studies) has shown to be a successful way to conduct a Primate Conservation Education program.

Key words: Arts for education, Conservation Education, Drawings analysis, Holistic experience, Mexico, Primates

Introduction

Science and Art are not opposed activities, they are human concepts related to the creativity processes that are required to generate them. The ability to imagine the unimaginable is a prized attribute for both artists and scientists. *Education on arts* is an education focused on professional and vocational guidance for a specific art [1], while *education through arts* considers art as a learning vehicle for other subjects and as a means to achieve more general educational outcomes [2]. Research advocates that integrating Art and Science education could engage learners in creating ingenious projects and inspire them to articulate science in different ways [3]. Nevertheless, the integration of arts in everyday science teaching is little known, especially in the field of biodiversity conservation [4]. Here, we present a case study of primate conservation at formal schooling in Southeastern Mexico from the approach of education through arts.

Primate conservation

Primates play an important role in structuring tropical forests, and accordingly, are often described as ecosystem engineers [5]. Frugivorous species are particularly important for maintaining ecosystems through ecological networks such as seed dispersal [6] alternatively, folivorous species make up most of the primate biomass in tropical forests [7]. Nevertheless, 63% of all primates are threatened with extinction [8], with the primary threats being tropical forest destruction, capture for the pet-trade, bushmeat hunting, pest control, injury due to infrastructure expansion, pollution, and disease [9]. For these reasons, primate conservation has become a prior concern for biological conservation [10].

In general, primates are important in many aspects of human societies, playing significant roles in cultures, religions, and even livelihoods; as well as being essential in human evolution, biology and ethology research [10]. Additionally, these animals are charismatic species, which

facilitates the implementation of conservation strategies by the local communities, and the general population [11, 12, 4], helping to reach further and wider goals in species conservation. However, the strategy of charismatic and flagship species will be more effective if local knowledge and perceptions are considered, and if the target species have links to their cultural identity [11]. Primates are particularly attractive to children; albeit unintentionally, children prefer animals with anthropomorphic features [13].

Mexico is the northernmost distribution for New World Primates with two of their native species *Alouatta pigra* and *Ateles geofroyi* categorized as ‘Endangered’ by IUCN [14]. The principal threats to these animals are deforestation and hunting for either food or pet trade [10].

Additionally, primate conservation programs take place under different socio-economic conditions such as high poverty rates, limited funding resources, some negative cultural stereotypes, and political instability and corruption [15]. In the face of these challenges, successful primate conservation requires a multidisciplinary approach that needs to be nourished by theory and practice from, at least, the fields of biology, anthropology, psychology, economics, and education [16]. Integrating the natural and social sciences will guarantee that decision-making during planning, implementation and management are guided by the best available information [17]. Conservation efforts should also encourage local participation and incorporate local knowledge systems to inform culturally-relevant educational programs that instill respect for primates and their habitats in culturally-relevant ways [18,19]. Furthermore, as a component of multidisciplinary conservation efforts, appropriate education and outreach programs can promote sustainable behavior, decline poaching levels, and guide the decision and policy making that impact the biodiversity and natural resources [20].

Conservation Education and Arts

Conservation Education (CE) is a crucial component in the process of solving current environmental problems through its role in increasing awareness and modifying attitudes of general population. As well as promoting the gaining of knowledge and skills in conservation researchers and practitioners [21]. Nevertheless, there is a need for seeking an interdisciplinary CE in order to be able to tackle complex, multi-disciplinary environmental challenges. To design a CE program, it is necessary to consider age, different learning strategies, and promote meaningful and transformative learning [22], In Mexico, CE has been mainly dominated by a traditional education perspective because of the influence of the fields of biology and technology, whereby environmental information is transmitted through teaching methods that generate receptive and passive learning, reinforcing a single area of human development: the cognitive domain [23, 24]. But the emotional domain is needed for the integrated child development (e.g. creativity, critical thinking) and for the harmonious relation with nature [22]. In addition, many children seem to have lost enthusiasm in nature because they perceive it less attractive than social media or electronic games. Hence, we need to seek for innovative habits to (re)awaken and (re)nourish the sensibility of children and re(build) a new relation with outdoors [25].

The emotional perspective on sustainable behavior needs to be included on the level of model design, as well as on the practical level of intervention programs to result in long-term changes in feelings and behaviors [26] For this reason, artistic and creative approaches can facilitate affective knowledge, as well as deepen the emotional connections between people and places [27], thereby maximizing all senses (auditory, visual, kinesthetic, etc.). This is particularly important for biological conservation, because applying these principles to conservation issues can foster both closer relationships with nature, and more creative solutions to certain issues.

Additionally, central components of the decision-making process are the emotions and the conflict that we can experience between the heart and the mind while making the choice. The skill to manage emotions and moral issues is important in the argumentation processes of science emphasized in science topics related to socio-scientific issues, conservation topics and sustainable development [28].

In addition, it is vital to work this topic in formal education, where the school becomes the cornerstone in promoting values about the society-environment relationship and fostering a critical spirit to face the different situations of environmental problems. Besides, in the Mexican and rural context, the school is a central point of connection with the rest of the community where children interact with teachers, maintenance personnel, parents, etc. The future decision-making group is in the classroom today and the current decision-makers have relatives at the school too.

On the other hand, the benefits of arts education in elementary education are numerous, such as strengthening self-esteem, stimulating creativity and learning, and other moral values that are not acquired with traditional subjects. However, in Mexico there is a weak presence of arts at the schools. From the 800 hours of classes that are taught annually in primary school, only 40 hours are dedicated to Arts subject [29,30]. A precise assessment of CE programs outcomes would provide accountability and identify specific the role that arts can play in biodiversity conservation. Conservation issues can only be solved with creative and critical thinkers, different ways of perceiving and caring about the world should help us to conserve it [22].

Primate conservation education

CE programs has been reported to change people’s perceptions, knowledge and behaviors; thus, they are considered a key element of primate conservation initiatives [16]. Nonetheless, at

indigenous contexts it is important to consider different approaches from the post-development concepts (e.g. buen vivir, ubuntu) in order to take into account the traditional knowledge and different worldviews and belief-systems [31].

In practice, primate CE programs are varied, facing several difficulties depending on the social and cultural context, encompassing different audiences, having dissimilar lengths of time, and employing a variety of methods including active and passive learning strategies such as nature clubs, documentaries or comic books [32, 33, 34, 35]. Despite there being recent projects incorporating some artistic activities in primate CE, systematic evaluations of effectiveness of these activities remain few or are based mainly in the verbal-domain such as questionnaires as opposed to drawings analysis or photo elicitation [17]. Moreover, in Mexico, there is an overall lack of evaluation of primate CE programs, none of which use artistic approaches. This study seeks to fill this gap by employing an arts-based approach to primate conservation education and systematically evaluating its effectiveness. In this paper, we designed and conducted an arts-and-science based educational program at formal schools from Southern Mexico in order to evaluate the effectiveness of different teaching strategies in conveying the importance of the black howler monkey (*A. pigra*).

Material and methods

Study site and subjects

We conducted the study in different communities selected randomly throughout Southern Mexico (Fig. 1) during an annual elementary school cycle (2015-2016).

Fig 1. The area where black howler monkeys are distributed and the communities that were randomly selected.

The 12 communities (6 rural and 6 urban), within Natural Protected Areas. 1: Celestún, 2: San Francisco Tinum (Yucatán); 3: Dzoyolá, 4: Punta Allen, 5: Bacalar (Quintana Roo); 6: Xpujil, 7: Champotón, 8: Puerto Rico (Campeche); 9 Redención del Campesino, 10: Playas del Rosario (Tabasco); 11: Jerusalen, 12: Nueva Palestina (Chiapas). * Digital cartography used in this Figure is distributed under a Creative Commons Attribution-Noncommercial 2.5 license from Geoportal Conabio.

The three Mexican primates species can be found in this area, study sites were selected based on the geographical distribution of the black howler monkeys (*A.pigra*) that corresponds to approximately 250,000 km^2 in the states of Tabasco, Chiapas, Campeche, Yucatan, and Quintana Roo). Communities were randomly chosen from INEGI database [36] with selection criteria being rural or urban (less or more than 2,500 people, respectively) and residence within and outside of protected areas (PAs). This area is characterized by a strong indigenous heritage, there are around seven million Maya people that still live today in Mexico and Guatemala, many of them are native speakers of Mayan and their variations (e.g. Ch'ol, Tzeltal, Tzotzil, Yukatek) rather than Spanish [37]. From this context, a total of 229 students aged 8-10 years from 12 primary schools participated in this study. We considered three variables to analyze in this paper: gender (boys 48.9% vs. girls 51.1%), context (rural 48.9% vs. urban 51.1%), and PAs (inside PAs 43.7% and outside PAs 56.3%). Our educational intervention was done with the official permission of the Secretaría de Educación Pública- Secretariat of Public Education in the Government office of each State, the School Council and the Municipal Comissioner.

Intervention design

For conducting and evaluating the arts-and-science based educational program at the selected schools we followed a sequential intervention that consisted in three stages at each school : 1) Rapport building phase (via ice breaker games and creating a climate of trust) as well as pre-evaluation of knowledge and perceptions through content drawing analysis [18], 2) Intervention (four groups at each school participated in arts-based education techniques, three of which were part of the intervention, with the 4th group serving as the control group); 3) Post evaluation of knowledge and perceptions (through drawings analysis), and reinforcement of learning one month after the intervention. In parallel, meetings were held with the parents and teachers to explain the activities that we had planned with kids (*see* Table 1). Interviews were also conducted in the community to understand the social and environmental context of schools.

Table 1. Description of all the techniques used detailing the focused sense, previous activities and materials used.

**Technique and sense**	**Previous activity**	**Main activity**	**Material**
**Storytelling: Auditory**	Listening to environmental sounds and recreating them	Storytelling performed by the artistic facilitator, with some sounds through speakers. Students were blindfolded to enhance listening	Speakers and scarfs
**Shadow puppets: Visual**	Guiding dog game and observational games of the natural environment	Vision was the predominant sense in this activity, where the story was told through shadow puppets performed by the artistic facilitator, supported	Homemade wooden puppet theatre with wheels,

		by text posters and sometimes accompanied with environmental sounds	styrene puppets and speakers
**Theatre: Kinesthetic**	Physical and theatre warm-up games	First reading the story loud together and ensuring their comprehension. Then, performing the story using only their imagination, body language and props	Story printed for team work and props (balls and scarfs)
**Control group: Mix**	Physical warm up games and a dance activity while music was playing	Future scenario activity about their community. Children were asked to draw a specific place of their community in the present and 50 years in the future	Sheet of paper and colored pencils

Arts-based Intervention

The intervention was carried out following different artistic languages, in which everyone was focused on a specific sense (Table 1). There was a main activity with three experimental groups: Storytelling (auditory sense), Shadow puppet (visual sense) and Theatre (kinesthetic sense). Each technique had the same duration (20-30 min), and the same content about black howler monkeys (geographical distribution, basic behavior, ecology, conservation, and worldview and traditional knowledge), but varied in the form or style in which it was communicated. Activities held for the control group were not related to black howler monkeys. Instead, a future scenario drawing activity was carried out, where children were asked to draw their community at the present and what it would be like in 50 years.

Previous activities were done with each group before the main activity, in order to prepare the artistic language that was going to be used (auditory, visual and kinesthetic). After the main activity took place, the facilitator talked to each team about their perceptions, and to find out what they understood about the activity. Finally, we gave the children an opportunity to express one of their favorite's scenes through a drawing. This activity was optional.

After the all the activities were finished, a *satisfaction survey* was used to identify children's perceptions and suggestions about our performance. Each participant was asked to answer eight questions about the program (i.e., What did you learn? Could you give your opinion? Do you think this topic is important? Did you like how we told you the story? Would you repeat the experience? Did you have enough time to learn? Did you have fun? Did you like working as a team?) To answer them we used a dartboard prototype with three different colors of codification: green – very satisfied, yellow – medium, and red – not satisfied. Also, we hung a board on the wall with the title "How did I find the experience" and encouraged the children to express their thoughts and feedback about the activities, our presence, or a special moment they wanted to share (Fig. 2).

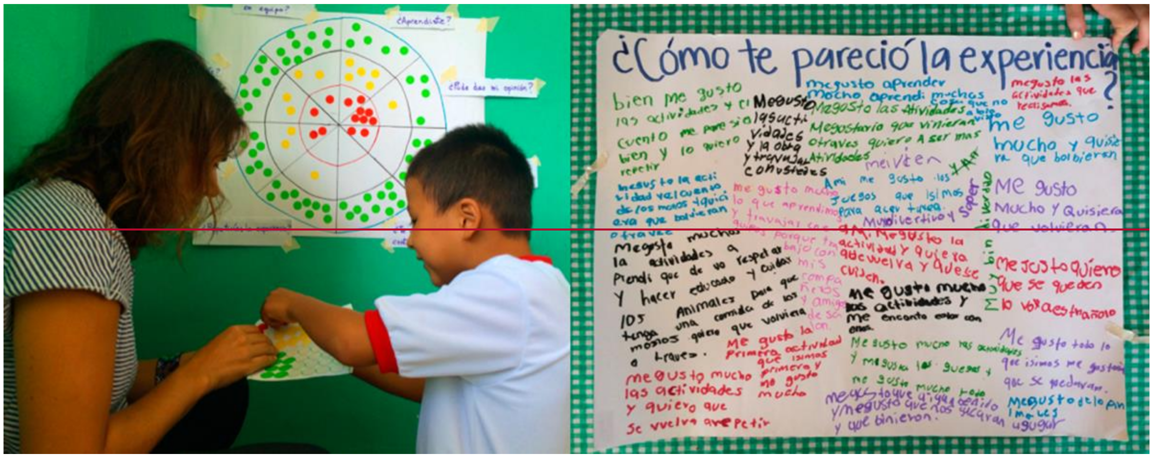

Fig. 2. *Left* A participant assessing our performance through the dartboard of what he likes and does not like. *Right* Space for kids to provide feedback on the activities.

Evaluation technique: drawings

Drawings were used for an evaluation method of children's knowledge and perceptions about black howler monkeys (Pre and Post) [38, 39,18]. A sheet of paper with a howler monkey silhouette was given to each child, crayons were provided and they were encouraged to draw to answer the following question 'What does this animal need to live well?' (Fig. 3). There was no discussion before starting the drawing session, except to introduce the activity. Students were given 50-60 minutes to complete the drawings.

Fig. 3. Some of the children responses through drawings, each drawing is unique to each child.

We asked teachers to not interact with children, discuss their drawings or provide additional explanation during the activity. Also, no books or images were allowed for the activity. After the drawing was completed, we asked a series of questions to clarify the objects and actions depicted and to gain an understanding of the children's perceptions. To minimize bias in the interpretation, each drawing was analyzed by a pair of researchers from different disciplinary backgrounds (i.e., conservation biology and psychology) but who were familiar with the monkeys and the children's culture. Both researchers took part in the classroom activities. Strict pre-determined rules of interpretation were followed (*see further information about the methodology at Franquesa-Soler et Serio-Silva* [18] Inter-rater reliability was measured with a percentage of agreement, which takes into account the chance agreement of two observers. The two evaluators had 83% of agreement rate, and a rate of 80% is considered reliable (40).

Data analysis

Drawing activity was used to evaluate the effectiveness of each method of intervention (storytelling, shadow puppets and theatre) in expanding students' knowledge about black howler monkey behavior, ecology, and conservation. We assessed students' knowledge about black howlers in terms of the following categories: 1) howler pelage color – with black being the correct color, 2) tree - at least one tree appears in the drawing, 3) canopy - locating this animal on a tree or branch), 4) food - any type of correct food source they can imagine this animal needs), 5) family - other howlers in the drawing, and 6) other - e.g., representations of other activities such as playing, sophisticated source of food, conservation messages. For each of these categories, we assigned a knowledge score of 0 (absence) or 1 (presence). Pre and Post evaluation were considered to evaluate the effectiveness of each method of intervention. In addition, specific indicators of the change between the Pre and Post were considered. Yellow mombin (*Spondias mombin*) also known locally as *jobo*, is a fruit from the Black howler monkey's diet and it was included in the content of the stories, so we evaluated the new presence of this element in the Post-drawings. We also assessed whether several assumptions or misconceptions that were detected in the Pre-analysis (*banana* as food source, *brown color* to describe this primate species, and locating the animal on the *forest floor* [18]) persisted following the interventions.

We used linear mixed models (LMMs) to analyze children's knowledge (scored as 0-6), using context (rural and urban), gender (boys and girls), location related to PAs (living inside or outside), state (Tabasco, Chiapas, Campeche, Yucatan and Quintana Roo), time (Pre and Post) as fixed factors and ID (each individual child) as a random factor in our models. For all models, appropriate error distributions were specified following Zuur et al. [41]. To determine the best model to assign to the dependent variable (score of children's knowledge) we used the Akaike's

information criterion (AIC). We performed LMMs with the R package nlme (Pinheiro et al., 2014) within the statistical program R (version 3.2.0) [43]. We calculated Increase of Learning (IL) representing the difference between the Score of Pre and Post intervention. We used repeated measures ANOVA (Bonferroni adjustment for multiple comparisons), and dependent T-test to make comparisons between pre- and post-evaluations. We also used independent T-tests and Kruskal-Wallis tests to compare between groups [44]. The α-level for statistical significance was set at 0.05. Finally, to assess students' satisfaction with our interventions, we calculated percentages for each level of satisfaction (i.e., green – very satisfied, yellow – medium, and red – not satisfied) from the dartboard exercise.

Results

From the LMM analyses, the best model indicated that the score of children's knowledge was predicted by PAs, Time (Pre and Post intervention), and the interaction between Technique and Time (Table 2); but not by Gender (male/female; LMM: P =0.0852) or Context (urban or rural; LMM: P = 0.1019).

Table 2. LMM analysis of factors affecting the Score of children's knowledge

Predictor variables	DF	*F*-VALUE	*P*-VALUE
**Protected Area (PAs)**	1	2.85	0.0930
**Time**	1	80.65	<0.001
**Technique:Time**	6	2.73	0.0140

df, degrees of freedom. AIC = 1451.209

We found differences in children's knowledge between Pre and Post evaluation for all techniques (*P*<0.01, *P*<0.05, *P*=0.01, respectively; Fig. 4), except for the control group (*P*=0.212; Fig. 4). Storytelling had the highest score from the Post-evaluation (3.9), followed by Theatre and Shadow Puppets, which shared similar scores (3.8 and 3.5, respectively). The storytelling technique produced a higher Increase of Learning (IL), or the difference between the Score of Pre and Post intervention (*See* Table A.2).

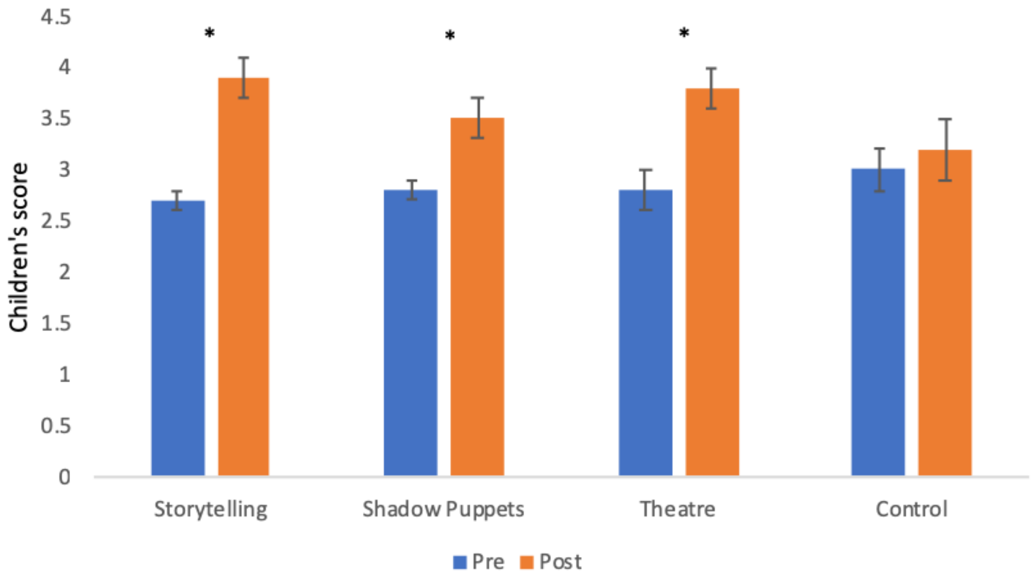

Fig. 4. Differences of children's learning between pre-and-post evaluation at each technique expressed in Score's mean, N= 229.

Context: Protected Area

Prior to the science-arts based intervention, children's knowledge differed significantly depending on whether they were living inside or outside the PA (3.1±0.1 vs. 2.7±0.1, respectively; t= 24.6; *df*= 1; p<0.01). However, following the intervention, we found no significant differences in children's knowledge by location (3.8±0.2 vs. 3.6±0.1, in and outside

the PA, respectively; t= 31.6; *df*=1; *P*=0.181). We did detect a higher Increase in Learning (IL) for those children who live outside the PA (0.87 vs. 0.68). For children living inside the PA, we found differences in knowledge in relation to the intervention technique [X^2(3, N = 89) = 20.39, *P* < 0.001]. Namely, the technique that increased knowledge the most was theatre (60.47%), followed by storytelling (54.94%). Knowledge generated from the shadow puppet (39.28%) did not differ from the control (28.48%).

Special indicators of the drawings

a) *Spondias mombin*: specific item from the black howler monkey's diet

Jobo (*Spondias mombin*) appeared for the first time in the post-intervention evaluation; it was present in 53.7% of the drawings and was influenced by the communicative technique that was used (X^2 = 32.859; *df* = 3; *p* <0.001). More children (67.9%) with the theater technique included the Jobo in their drawings as part of the diet of howler monkeys.

b) *Decrease of Assumptions*:

In addition to the IL produced between the Pre and Post intervention, some assumptions depicted from Pre-analysis [18] have decreased too (Table 3), such as including less *bananas* for food or drawing less times black howlers at the forest floor. Differences were not found among techniques.

Table 3. Differences between Pre and Post evaluations regarding the most assumptions detected in the Pre-analysis.

Assumption	%Pre	%Post	X^2	*df*	Pvalue
**Banana**	69.1	30.9	61.72	1	<0.001
**Forest Floor**	59.8	40.2	34.45	1	<0.001
**Brown color**	57.8	42.2	15.91	1	<0.001

Satisfaction survey

Regarding the evaluation of children’s feedback about the CE program, a majority of positive answers were obtained in all the questions. Broadly, more than 70% of the children answered that they felt very satisfied in six of the eight questions. Questions with the highest percentage of positive answers were about: whether they learnt something new about black howler monkey conservation (72%), the importance of the topic (84%), their enjoyment during the process (88.8%), and the techniques used (86.6%). The questions scored negatively included the duration (8% not satisfied and 24.9% medium) and expression their opinions (15.8% not satisfied and 25.2% medium) (Fig. 5).

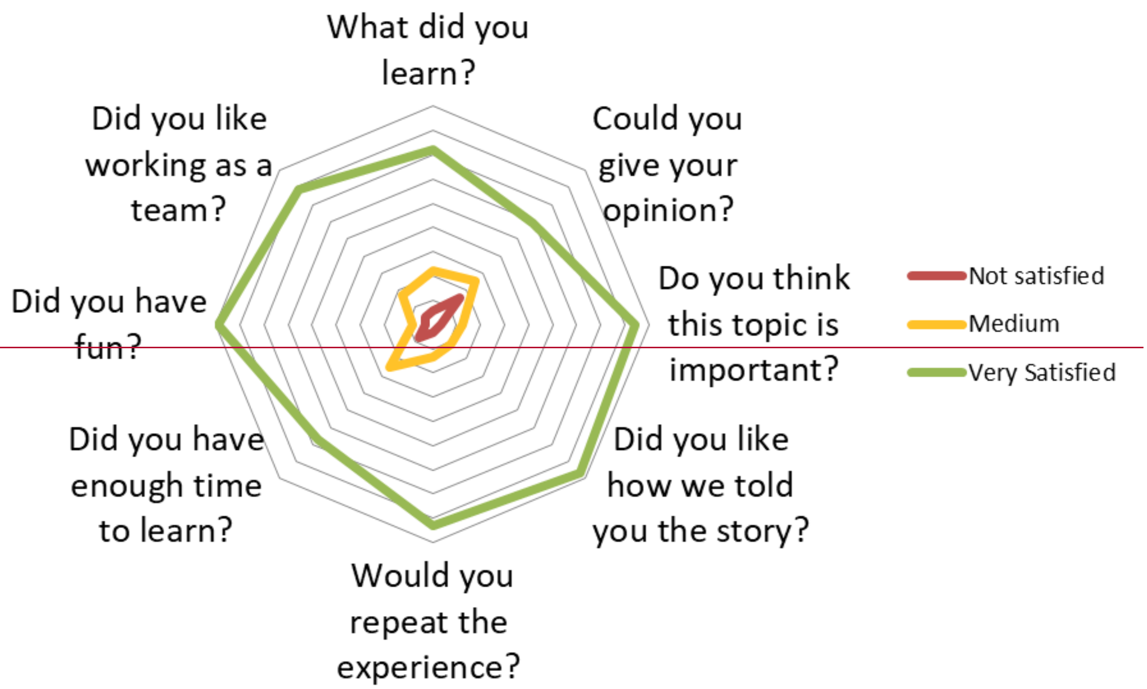

Fig. 5. Children’s feedback through answering eight questions about our performance, n=224

In order to obtain a further understanding about children’s perceptions of and reactions to the program, we also took into account the messages from the board (Fig. 2). Most of the messages

(71.3%) were about what they liked more. Particularly, they were referring to some humorous or positive moments of specific activities, including some of the icebreaker games (e.g. "I liked painting, working with you, dancing and singing, I liked everything"; "I liked to perform the theatre, I had a lot of fun and I learnt a lot"; "I liked when we saw the show of the monkey and the boy" or "I liked the shadow puppet"). Generally, most of the messages (65.2%) were related to positive feelings and emotions, and some messages were requests to continue our work with them ("I liked doing activities with you, I hope you come back"). Some sentences (40.6%) were linked to the black howler monkeys and what the children learned about them ("I liked to perform the theatre and to learn about monkeys, and to draw them").

Discussion

Quality of Education and Conservation programs: the need for innovation with methodologies

Our study examined the effectiveness of different arts-based pedagogical techniques in improving the message for the conservation of primates and their natural habitat. Our aim was to find the best ways to have a positive educational experience with participatory and inclusive methods, as well as paying special attention to the emotional connection between children and nature or, in this case, the black howler monkeys. In Mexico, Education for Sustainability (EfS) is still relegated in the school curricula [23]. Therefore, our work supports an alternative when implementing EfS projects in formal schooling.

Regarding to the content of these programs, in some Mexican schools there is a good deal of environmental education about recycling and water care, but this is not directly related to how this knowledge can benefit local needs, biological conservation, and land use [32]. There is a

need for holistic approaches, where all environmental contents are linked to individual, collective and global trends and lifestyles.

Hence, from our experience we identified a gap in the teaching field of EfS in Mexico: the species conservation domain. Additionally, it seems that children are only following rules instead of being involved in meaningful projects [38]. With the arts-based education approach we were able to encourage critical thinking, as well as promote responsible actions based on autonomous decisions. Working on Primate Conservation allowed for the establishment of real connections between primate conservation issues and local community issues in ways that are not easy to teach through traditional methods. In Mexico this model is still common, where educators -many times with limited resources for doing their job -simply hand down knowledge and students do not play an active role in the learning process [23,45]. The components of choice, responsibility, simplicity participation in decision-making and feedback with results that are integral to environment-based education have been used in many different fields to empower learners, engage participants, improve training, and create appropriate development projects [46].

PAs and technique predicted children's score about the black howler monkeys

We found that children's knowledge about black howler monkeys was predicted by the technique used (storytelling, shadow puppet, and theatre) related to the moment of intervention, and PAs [inside or outside the Protected Area (PA)]. In contrast to other studies, children's knowledge score was not predicted by gender [47, 48, 49] or by context (i.e., rural vs. urban areas). Other studies have provided evidence of gender impacts on pre-program knowledge, often with males showing improved knowledge. We had similar results in the Pre-evaluation, where in some categories boys had more knowledge about black howler monkeys [18] but with the implementation of the program these

differences disappeared. This finding supports the importance of designing projects, such as this one, that consider the inclusivity of gender by providing spaces where girls can express and learn the same way that boys do. This design feature is important in Mexico, where gender inequalities still exist, especially in rural communities [50]. It is also particularly significant for CE because positive associations between gender equality and sustainable development have been demonstrated in countries such as Nepal and India [51].

Previous studies have found differences in knowledge about biodiversity depending on the place of residence [52] where urban residents showed greater concern [53, 54]. Other recent studies, conversely, indicate that differences among rural and urban citizens may be weakening. The reason of factors influencing on reducing differences between rural and urban populations have been linked to (1) increase of environmental services for rural areas [55] (2) migration of urban citizens with positive environmental values to rural communities [56], and the decrease of economic dependence related to natural resources industries by rural communities. In a related study to the present one, we found differences in the Pre-evaluation [18], but in a reverse pattern: rural children knew more about these animals because they had more opportunities to see them. However, in the post-evaluation of this study we found that *context* (urban or rural) was not a factor influencing children's knowledge, thereby confirming again that the project worked for both contexts and reduced the differences in conservation knowledge between rural and urban areas.

Technique used

Except for the control group, all artistic techniques (storytelling, shadow puppets and theatre), increased children's knowledge score about the black howler monkeys. These findings confirm the general success of the arts and science based intervention. It is important to assume that CE

programs do not work everywhere in the same way. Accordingly, it is important to test different techniques to include cultural factors, gender, and different learning styles. For instance, applying education techniques (including evaluations) that work for a Western society audience might not necessarily lead to the expected change in a developing country [4].

In Mexico, EfS is poorly implemented in the school curricula, and Arts education is rarely taught [30]. The Arts allow children to learn through all senses and promote the use of multiple intelligences [20]. Additionally, in Southern Mexico there are roughly 16 indigenous languages [58], and while the majority of schools are not indigenous, there are language, reading and writing difficulties. The underlying problem is that, since Mexico is culturally and linguistically diverse, the curriculum is unique for the whole country and the assessment instruments are in Spanish [59]. Also, teaching is typically rote-learning (memorization of information based on repetition) instead of educator-learner interactions with creative education techniques [60,61]. Hence, before applying and evaluating an education program it is crucial to adapt the program contents and the type of evaluation question to the local context, as well as considering a universe of different learners [49].

Although all techniques successfully increased children's knowledge, *Storytelling* stands out as being the technique that produced both a higher Score and Increase of Learning (IL). This finding matches with the results found in the Pre-program, when learning preferences were evaluated in the same group of children [45]. The Pre Program study revealed that auditory was the preferred learning style of the children (46.4% of the sample), in accordance to Dunn & Dunn [62] and Barbe & Milone [63], who reported that it is common for children at the primary level to learn and retain information through the auditory sense. In this case we wanted to design tools which considered their preference, invited them to reflect and participate actively, and used a

technique that was important for the Mayan cultural context [64]. Furthermore, current studies show how narrative and storytelling can facilitate the communication of science to nonexperts [65], expanding the context of “framing” as being an important component of public outreach [66].

Stories form a link between our imagination and our environment [67]. In fact, for most cultures (e.g. Mayan culture), oral traditions and stories convey local knowledge and wisdom about the environment and our relationship with the earth and with others [64]. Narratives allow the audience to get to know the characters, see different perspectives, and experience their emotions and their environments. Furthermore, it is a good methodology to achieve emotional connections and symbolic thinking, goals which technology sometimes cannot accomplish. For example, replacing free play and storytelling with the audiovisual system undermines the symbolic-metaphorical intelligence of children [68]. Excessive audiovisual activities isolate children from both the world of imagination and the natural environment. However, during free play, theatre, storytelling and similar activities, children can develop inner images. This ability is the foundation of future symbolic and metaphorical thinking, and concrete operational thinking, mathematics, science, philosophy and all forms of knowledge considered as higher education [68].

Our study shows that if multiple intelligences and senses are considered, it can improve the effectiveness of CE programs. Not only for the part of the intervention, but the evaluation should also be inclusive for all learners and indigenous cultures, since most times in primate CE assessment is done through questionnaires [4,61,34]. The drawings have been excellent tools for evaluating the effectiveness of CE programs, as well as an artistic approach that evaluated the knowledge of these animals. If it is an education based on novel tools that promote other emotional processes, then drawing is in accordance with this approach.

Protected Areas and non-Protected Areas

Differences in knowledge between those living inside and outside of PAs were found during the Pre-evaluation [18] but they disappeared when Post-evaluation was analyzed. This finding suggests that, as we found with the other context variable (rural vs. urban), that the project worked for both children living inside or outside PA and reduced differences between these groups. *Theatre* was the technique which was most effective inside PAs, and this could be due to the one specific school inside of a PA that showed an incredible excitement for this technique. Our results do not support the idea of using only this technique inside PA, but more studies are needed to test different methodologies for different situations and contexts, and by considering the diversity of learners.

Learning indicators, decrease of misconceptions

During the Pre-program evaluation, the more common misconceptions or assumptions around children's understanding about black howler monkeys were detected [18]. First, we used *Spondias mombin* as an indicator of knowledge. Then, to analyze if misconceptions decreased, we selected the three most common confusions depicted from the Pre-evaluation: *banana* as a food source, *brown color* for describing this animal, and locating them on the *forest floor*. Jobo (*Spondias mombin*) was considered as an indicator of knowledge because it is one of the tree fruit species most consumed by these animals [69]. Long-term studies reveal that black howler monkeys have a highly varied diet, largely dependent on the availability of preferred foods [6]. Therefore, it was selected for being representative both to the black howler monkey diet and to the Southern Mexico region. During the Post-evaluation, *jobo* appeared in more than half of the drawings (during the Pre-test none), indicating that the intervention was successful, that the story was understood and that the children learned about one of the elements of the actual diet of black howler monkeys.

Regarding the technique used, *theatre* was the most effective technique for children incorporating *jobo* in the drawings and, at the same time, even reducing the misconception of *bananas* being black howler foods. It is important to not only show that some elements are not part of their diet, but also to show which elements are correct. The same principle applies with other pro-environmental behaviors, as it does not help to say what should and should not be done, but show in a simple way what can be contributed [70]. During the *theatre* activity, children played with props, sometimes simulating the *jobo*. They integrated it for the theatre play, they ate the jobo and defecated it to represent the role of black howler monkeys as seed dispersers. The action of touching and using it in a specific situation might serve to materialize and internalize it as a *concrete manipulative* [71]. According to Carbonneau et al. [71], concrete manipulatives make easy learning by (a) promoting the abstract reasoning [72], (b) enhancing thought-provoking learners' real-world knowledge [73], (c) allowing the learner to apply the concept for improved encoding [74], and (d) offering chances for learner-driven exploration while discovering new concepts [75].

With respect to *assumptions* or *misconceptions*, these three decreased in the Post-test analysis, indicating that our intervention not only helps increase learning, but also to clarify some children's confusions. The origin of the myth that monkeys naturally eat bananas remains unidentified, but its persistence is a misconception that primate conservation education programs should address as it tends to anthropomorphize monkeys. The *banana* concept was tackled through the help of "dislike" sounds and negative expressions during the interventions. The *brown color* was explained in the stories with the different formats, but it was also reinforced by showing a real picture of the animal at the end of the activity. The *forest floor* misconception was highlighted in the story by relaying that howlers arrived at an extremely deforested area and

needed to cross the highway on the ground to access to other sources of fruit, but that some died while trying. Other threats and diseases that these animals face in the *forest floor* were also shown.

Satisfaction survey

The use of evaluation tools is really important for conservation education programs [61]. To know which are the most effective conservation programs, it is essential to assess the children's perceptions and knowledge about certain topics. It is also important to understand the children's feelings and opinions about the conservation program [4, 34]. We sought to assess the latter through the *satisfaction survey*. Because children from Mexican communities are usually taught through the traditional teaching system, it is not common for them to be consulted about the educational programs that they receive or to be asked to evaluate their teachers' performance. For this reason, it is important to take into account that their answers may be conditioned, resulting in overestimating our performance. However, the satisfaction survey is still a useful tool to explore their reactions about our activities and also to enhance critical thinking, which is necessary for the decision-making processes about sustainability issues.

The general results of the satisfaction survey show a positive response to this conservation education program by the students. Children stated that they had learned about the black howler monkey conservation and considered it an important issue. We attribute these reactions to their enjoyment of the learning process and the arts-based techniques used. Including an emotional component in the design of education programs can act as a motivation factor by facilitating students' engagement and making the learning process more stimulating [26]. Although the percentage of negative perceptions obtained in this survey was very low, it is important to consider that some children thought that the time invested in the implementation of the program

should have been longer. This is in agreement with some primate education projects that have found that longer programs are associated with a greater increase in participants' knowledge [61,33] and that long-lasting projects would be more effective (Swartz et al. 2012). That said, other studies found that the length of participant involvement did not affect knowledge retention [16,76]. The message posts left by the children on the board all contained positive content. Most of these messages allude to their enjoyment with some of the artistic activities or icebreaker games we did during the process. On the one hand, the use of games is a powerful teaching strategy because it makes the learning process more interesting and fun [77]. On the other hand, researchers have found that arts offer a way for people to connect emotionally to the conservation topic of interest, and therefore are proving successful. A good learning process includes feelings and is vital to achieving long-term changes in perceptions and behaviors [26]. Moreover, children showed in their messages that they really appreciated our activities and the time they spent with us, and that they would love to repeat the experience.

Conclusions

We contend that the program was effective not only because of the techniques used, but because it was a holistic program. According to Stern et al. [78], after reviewing 86 programs, they found that a common element of success in the interpretation of Education for Sustainable Development field was the concept of providing a holistic experience [78]. Since we started asking for permits from the *Secretaría de Educación Pública* - Secretariat of Public Education in the Government office of each State, we considered presentation days, ice breaker games, establishing rapport or trust with the school community (and the general community). We also did a pre-test with inclusive, qualitative and comfortable evaluating tools (drawings). We worked with small

groups, naming those groups with the corresponding fruits and animals which appeared in the story. We conducted activities beforehand in order to prepare the artistic language for the different techniques of the intervention. We based the program on an artistic approach, conducting several dialogues in between, and other drawing activities to reinforce learning. We also conducted the satisfaction survey, as well as games to invite reflection and the sharing of experiences, emotions and cultural exchanges. We spent 2-3 weeks at each place, living there, sharing food with the local people, getting to know the parents, grandparents and places in which they loved to play or explore after school. We also participated in some activities important for the local community (e.g. town or school festivals, sport events). To sum up, it was a holistic experience. Holistic experiences involve conveying a complete idea or story within the educational context. They thus carry high potential to provide a coherent picture of the relevance of the educational activity and a clear take-home point for students to reflect upon or pursue following the experience [78].

Acknowledgments

The authors are grateful to the participating schools. We would like to express our deepest gratitude to Erin P. Riley and Maria Lay for their valuable suggestions to the structure of the manuscript, language editing, and proofreading. Thanks to John Aristizabal, Esther Castro, Laura Jayme for their support during fieldwork activities, and Adriana Sandoval-Comte who helped with mapping. We thank Consejo Nacional de Ciencia y Tecnología (CONACyT, Mexico) for offering the scholarship to MFS (N° 556384), Instituto de Ecología, A.C (INECOL, A.C, Mexico), DGAPA-UNAM Postdoctoral Fellowship, and to JCSS for financial support.

References

1. Touriñán-López JM. Claves para aproximarse a la educación artística en el sistema educativo: educación “por” las artes y educación “para” un arte Estud Sobre Educ. 2011; 21:61-81.
2. Bamford A. The Wow Factor: Global Research Compendium on the impact of the Arts in Education. Germany, Münter: Waxmann; 2006.
3. Turkka J, Haatainen O, Aksela M. Integrating art into science education: a survey of science teachers’ practices. Int J Sci Educ. 2017; 39(10):1403-1419. https://doi.org/10.1080/09500693.2017.1333656
4. Breuer T, Mavinga FB, Evans R, Lukas KE. Using video and theater to increase knowledge and change attitudes–Why are gorillas important to the world and to Congo? 2017. Am J Primatol. 79(10): e22692 https://doi.org/10.1002/ajp.22692
5. Chapman CA, Bonnell TR, Gogarten JF, Lambert JE, Omeja PA, Twinomugisha D, ... & Rothman JM. Are primates ecosystem engineers? Int J Primatol. 2013; 34(1):1-14. https://doi.org/10.1007/s10764-012-9645-9
6. Pavelka MS, Knopff KH. Diet and activity in black howler monkeys (*Alouatta pigra*) in southern Belize: Does degree of frugivory influence activity level? Primates. 2004; 45(2)105-111. https://doi.org/ 10.1007/s10329-003-0072-6
7. Fashing PJ, Cords M. Diurnal primate densities and biomass in the Kakamega Forest: an evaluation of census methods and a comparison with other forests. American Journal of Primatology. 200; 50(2):139-152.

8. Cotton A, Clark F, Boubli J., Schwitzer C. IUCN Red List of Threatened Primate Species. In: Wich SA, Marshall AJ, editors. An Introduction to Primate Conservation. Oxford, UK: Oxford University Press; 2016 pp: 31-18.
9. Wilkie DS, Bennett EL, Peres CA, Cunningham SA. The empty forest revisited. Ann NY Acad Sci. 2011; 1223(1):120-128 https://doi.org/10.1111/j.1749-6632.2010.05908.x
10. Estrada A, Garber PA, Rylands AB, Roos C, Fernandez-Duque E, Di Fiore A, ..., & Rovero F. Impending extinction crisis of the world's primates: Why primates matter? Sci Adv. 2017; 3(1). e1600946. https://doi.org/10.1126/sciadv.1600946
11. Bowen-Jones E, Entwistle A. Identifying appropriate flagship species: the importance of culture and local contexts. Oryx. 2002; 36(2):189-195. https://doi.org/10.1017/S0030605302000261
12. Clucas B, McHugh K, Caro T. Flagship species on covers of US conservation and nature magazines. Biodivers Conserv. 2008; 17(6):15-17. https://doi.org/10.1007/s10531-008-9361-0
13. Morris D. El hombre al desnudo. Un estudio objetivo del comportamiento humano. Barcelona, Spain: Nauta; 1980.
14. IUCN. The IUCN Red List of Threatened Species. Version 2016–3. www.iucnredlist.org. Accessed 15 Dec 2017.
15. Fisher B, Christopher T. Poverty and biodiversity: measuring the overlap of human poverty and the biodiversity hotspots. Ecol Econ. 2007; 62(1):93-101. https://doi.org/10.1016/j.ecolecon.2006.05.020

16. Kling KJ, Hopkins ME. Are we making the grade? Practices and reported efficacy measures of primate conservation education programs Am J Primatol. 2015; 77(4)434-448. https://doi.org/10.1002/ajp.22359

17. Bennett NJ, Roth R, Klain SC, Chan KM, Clark DA, Cullman G, ..., Thomas RE. Mainstreaming the social sciences in conservation. Conserv Biol. 2017; 31(1):56-66.

18. Franquesa- Soler M, Serio- Silva JC. Through the eyes of children: Drawings as an evaluation tool for children's understanding about endangered Mexican primates. Am J Primatol. 2017; 79(12):e22723. https://doi.org/10.1002/ajp.22723

19. Dore KM, Riley EP, Fuentes A. Ethnoprimatology: A Practical Guide to Research at the Human-Nonhuman Primate Interface. Cambrige, UK: Cambridge University Press; 2017.

20. Jacobson SK, McDuff M, Monroe M. Conservation education and outreach techniques. Oxford, UK: Oxford University Press; 2015. https://doi.org/10.1111/emr.12251

21. Brewer C. Translating data into meaning: education in conservation biology. Conserv. Biol. 2006; 20(3):689-691. https://doi.org/10.1111/j.1523-1739.2006.00467.x

22. Jacobson, S. K., Mcduff, M. D., Monroe, M. C., 2007. Promoting conservation through the arts: outreach for hearts and minds. Conserv. Biol. 21(1):7-10. https://doi.org/10.1111/j.1523-1739.2006.00596.x

23. Barraza L, Ceja-Adame MP. La planeación y la realización de la educación ambiental. In: Sánchez O, Zamorano P, Peters E, Moya H, editors. Temas de conservación de vertebrados silvestres en México, SEMARNAT, INE, USF&WS. México; 2011. pp: 321-331.

24. Varela-Losada M, Pérez-Rodríguez U, Álvarez-Lires FJ, Álvarez-Lires MM. Desarrollo de competencias docentes a partir de metodologías participativas aplicadas a la Educación

Ambiental. Form. Univ. 2014; 7(6)27-36. http://dx.doi.org/10.4067/S0718-50062014000600004

25. Van Boeckel J. Arts-based environmental education and the ecological crisis: Between opening the senses and coping with psychic numbing. In: Drillsma-Milgrom B, Kirstinä L, editors. Metamorphoses in children's literature and culture. Turku, Finland: Enostone; 2009. pp.145-164.
26. Kals E, Maes J. Sustainable development and emotions. In: Schmuck P, Schultz WP, editors. Psychology of Sustainable Development. Boston, USA: Springer; 2002. pp. 97-122.
27. Adcock L, Ballantyne R. Drama as a tool in interpretation: Practitioner perceptions of its strengths and limitations. Aust J Env Educ. 2007; 23:31-44. https://doi.org/10.1017/S0814062600000690
28. Eilks I. Science education and education for sustainable development - justifications, models, practices and perspectives. EURASIA J Math Sci & Tech. Educ. 2016; 11(1):149-158. http://dx.doi.org/10.12973/eurasia.2015.1313a
29. Morton Gomez VE. Una aproximación a la educación artística en la escuela. México DF, México: Universidad Pedagógica Nacional; 2001.
30. Fernández A. La educación artística y musical en México. Incompleta, elitista y excluyente. Cuad Interam Inv Educ Music. 2003; 2(4):87-100.
31. Acosta A. El Buen Vivir como alternativa al desarrollo. Polít Soc. 2015; 52(2):299-330. http://dx.doi.org/10.5209/rev_POSO.2015.v52.n2.45203

32. Dolins FL, Jolly A, Rasamimanana H, Ratsimbazafy J, Feistner ATC, Ravoavy F. Conservation education in Madagascar: Three case studies in the biologically diverse island-continent. Am J Primatol. 2010; 72(5):391-406. https://doi.org/10.1002/ajp.20779
33. Wright JH. Use of film for community conservation education in primate habitat countries. Am J Primatol. 2010; 72(5):462-466. https://doi.org/10.1002/ajp.20749
34. Rakotomamonjy SN, Jones JPG, Razafimanahaka JH, Ramamonjisoa, B, Williams SJ. The effects of environmental education on children's and parents' knowledge and attitudes towards lemurs in rural Madagascar. Anim Conserv. 2015; 18(2):157-166. https://doi.org/10.1111/acv.12153
35. Leeds A, Lukas KE, Kendall CJ, Slavin MA, Ross EA, Robbins MM, Bergl RA. Evaluating the effect of a year-long film focused environmental education program on Ugandan student knowledge of and attitudes toward great apes. Am J Primatol. 2017;79(8):e22673. https://doi.org/10.1002/ajp.22673
36. Instituto Nacional de Estadística, Geografía e Informática (INEGI). Censo de Población y Vivienda, 2013 (Informe nacional y estatales), México; 2013. Retrieved from: http://www.inegi.org.mx/
37. Nations, JD. The Maya tropical forest: people, parks, and ancient cities. Austin, USA: University of Texas Press; 2006.
38. Barraza L. Educar para conservar: Un ejemplo en la investigación socioambiental. In: Barahona A, Almeida-Leñero L, editors. Educación para la conservación, Ciudad de México, México: Las prensas de ciencias, UNAM; 2006. pp. 255–276.

39. Pellier AS, Wells JA, Abram NK, Gaveau D, Meijaard E. Through the eyes of children: Perceptions of environmental change in tropical forests. PLoS ONE 2014; 9(8):e103005. https://doi.org/10.1371/journal.pone.0103005
40. Lehner PN. Handbook of ethological methods. Cambridge, UK: Cambridge University Press; 1998. https://doi.org/10.1086/420284
41. Zuur A, Leno EN, Meesters E. A beginner's guide to R. New York, NY: Springer; 2009.
42. Pinheiro J, Bates D, DebRoy S. Sarkar D. Core Team, 2014. nlme: Linear and nonlinear mixed effects models_. R package version 3.1– 117; 2009.
43. R Core Team. R: A Language and Environment for Statistical Computing. R Foundation for Statistical Computing, Vienna, Austria; 2016. https://www.R-project.org/
44. SPSS Inc. 2008. SPSS Statistics for Windows, V 17, 0. SPSS Inc.: Chicago.
45. Franquesa-Soler M, Barraza L, Serio-Silva JC. Children's learning preferences for the development of conservation programs in Mexican communities. J Educ Res 2018: http://10.1080/00220671.2018.1427038
46. Kaplan S. Human nature and environmentally responsible behavior. J Soc Issues. 2000; 56(3):491-508. http://dx.doi.org/10.1111/0022-4537.00180
47. Mulder MB, Schacht R, Caro T, Schacht J, Caro B. Knowledge and attitudes of children of the Rupununi: Implications for conservation in Guyana. Biol Conserv. 2009; 142(4):879-887. https://doi.org/10.1016/j.biocon.2008.12.021
48. Keane A, Ramarolahy AA, Jones JPG, Milner-Gulland EJ. Evidence for the effects of environmental engagement and education on knowledge of wildlife laws in Madagascar. Conserv Lett. 2011; 4(1):55–63. https://doi.org/10.1111/j.1755-263X.2010.00144.x

49. Borchers C, Boesch C, Riedel J, Guilahoux H, Ouattara D, Randler C. Environmental education in Côte d'Ivoire/West Africa: Extracurricular primary school teaching shows positive impact on environmental knowledge and attitudes. Int J Sci Educ. 2014: 4(3): 240-259. https://doi.org/10.1080/21548455.2013.803632

50. Bosco EB. Los límites de la escuela. Educación, desigualdad y aprendizajes en México. Ciudad de México, México: El Colegio de Mexico; 2011.

51. Tyagi N, Das S. Gender mainstreaming in forest governance: Analysing 25 years of research and policy in South Asia. Int Forest Rev. 2017; 19(2): 234-244. https://doi.org/10.1505/146554817821255132

52. Huddart- Kennedy E, Beckley TM, McFarlane BL, Nadeau S. Rural-urban differences in environmental concern in Canada. Rural Sociol. 2009; 74(3):309-329 https://doi.org/10.1526/003601109789037268

53. Fortmann L, Kusel J. New voices, old beliefs: Forest environmentalism among new and long-standing rural residents. Rural Sociol. 1990; 55(2): 214-232. https://doi.org/10.1111/j.1549-0831.1990.tb00681.x

54. Lowe GD, Pinhey TK. Rural-urban differences in support for environmental protection. Rural Sociol. 1982; 47(1):114-128. ç

55. Smith MD, Krannich RS. ''Culture clash'' revisited: Newcomer and longer-term residents attitudes toward land use, development, and environmental issues in rural communities in the Rocky Mountain west. Rural Sociol. 2000; 65(3):396-421. https://doi.org/10.1111/j.1549-0831.2000.tb00036.x

56. Saphores JM, Nixon H, Ogunseitan O, Shapiro A. Household willingness to recycle electronic waste: An application to California. Environ Behav. 2006; 38(2):183-208. https://doi.org/10.1177/0013916505279045

57. Jones RE, Fly MJ, Talley J, Cordell HK. Green migration into rural America: The new frontier of environmentalism? Soc Nat Resour. 2003; 16(3):221-238. https://doi.org/10.1080/08941920309159

58. Sánchez L. Mexican Indigenous Languages at the Dawn of the Twenty-First Century edited by Margarita Hidalgo. J Socioling. 2011; 15(3):422-425. https://doi.org/10.1111/j.1467-9841.2011.00498.x

59. Schmelkes S. Educación y pueblos indígenas: problemas de medición. Rev. Int. Estad Geogr. 2013; 4(1):5-13.

60. Bettinger TL, Kuhar CW, Lehnhardt K, Cox D, Cress D. Discovering the unexpected: Lessons learned from evaluating conservation education programs in Africa. Am J Primatol. 2010; 72(5):445-449. https://doi.org/10.1002/ajp.20735

61. Kuhar CW, Bettinger TL, Lehnhardt K, Tracy O, Cox D. Evaluating for long-term impact of an environmental education program at the Kalinzu Forest Reserve, Uganda. Am J Primatol. 2010; 72(5), 407-413. https://doi.org/10.1002/ajp.20726

62. Dunn R, Dunn K. Teaching Students through their Individual Learning Styles. A Practical Approach. Reston, USA: Prentice Hall; 1978.

63. Barbe WB, Milone MN. What we know about modality strengths. Educ. Leadership. 1981; 38(5), 378-380.

64. MacDonald MR. Traditional storytelling today: An international sourcebook. New York, USA: Routledge; 2013.

65. Dahlstrom MF. Using narratives and storytelling to communicate science with nonexpert audiences. Proc. Natl. Acad. Sci. 2014; 111(4): 13614-13620. https://doi.org/10.1073/pnas.1320645111

66. Martinez-Conde S, Macknik SL. Opinion: Finding the plot in science storytelling in hopes of enhancing science communication. P. Natl. Acad. Sci. 2017; 114(31), 8127-8129. https://doi.org/10.1073/pnas.1711790114

67. Caduto MJ, Bruchac J. Keepers of the Animals: Native American Stories and Wildlife Activities for Children. Colorado, USA: Fulcrum Publishing; 1997.

68. Pearce JC. Evolution's end claiming the potential of our intelligence. New York, USA: HarperCollins; 1993.

69. Aristizabal-Borja J, Pozo-Montuy G, Pérez-Torres J, Serio-Silva JC. Annotations on the feeding ecology of black howler monkeys in an overcrowded fragment in Balancán, Tabasco, México. Univ. Sci. 2011; 16(2):140-16 http://dx.doi.org/10.11144/javeriana.SC16-2.aotf

70. Pepper, C. Engaging People in Sustainability Daniella Tilbury & David Wortman. Gland, Switzerland and Cambridge, UK: Commission on Education and Communication, IUCN, 2004. Aus J Env Educ. 2005; 21:131-133. https://doi:10.1017/S0814062600001026

71. Carbonneau KJ, Marley SC, Selig JP. A meta-analysis of the efficacy of teaching mathematics with concrete manipulatives. J Educ Psychol. 2013; 105(2):380-400. http://dx.doi.org/10.1037/a0031084

72. Montessori M. The Montessori method. New York, USA: Schocken; 1964.

73. Baranes R. Perry M, Stigler JW. Activation of real-world knowledge in the solution of word problems. Cognition Instruc. 1989; 6(4):287-318. https://doi.org/10.1207/s1532690xci0604_1

74. Kormi-Nouri R, Nyberg L, Nilsson LG. The effect of retrieval enactment on recall of subject-performed tasks and verbal tasks. Mem Cognition. 1994; 22(6):723-728. https://doi.org/10.3758/BF03209257

75. Piaget J, Coltman D. Science of education and the psychology of the child., New York, USA: Grossman; 1974.

76. Evely AC, Pinard M, Reed MS, Fazey I. High levels of participation in conservation projects enhance learning. Conserv Lett. 2011; 4(2):116-126. https://doi.org/10.1111/j.1755-263X.2010.00152.x

77. Teed R. Game-based Learning. Starting Point-Teaching Entry Level Geoscience; 2004 Retrieved February 25, 2005, from http://serc.carleton.edu/introgeo/games/index.html

78. Stern MJ, Powell RB, Hill D. Environmental education program evaluation in the new millennium: what do we measure and what have we learned? Environ Educ Res. 2014; 20, 581-611. https://doi.org/10.1080/13504622.2013.838749

Figure 1

Click here to download Figure Fig1.tif

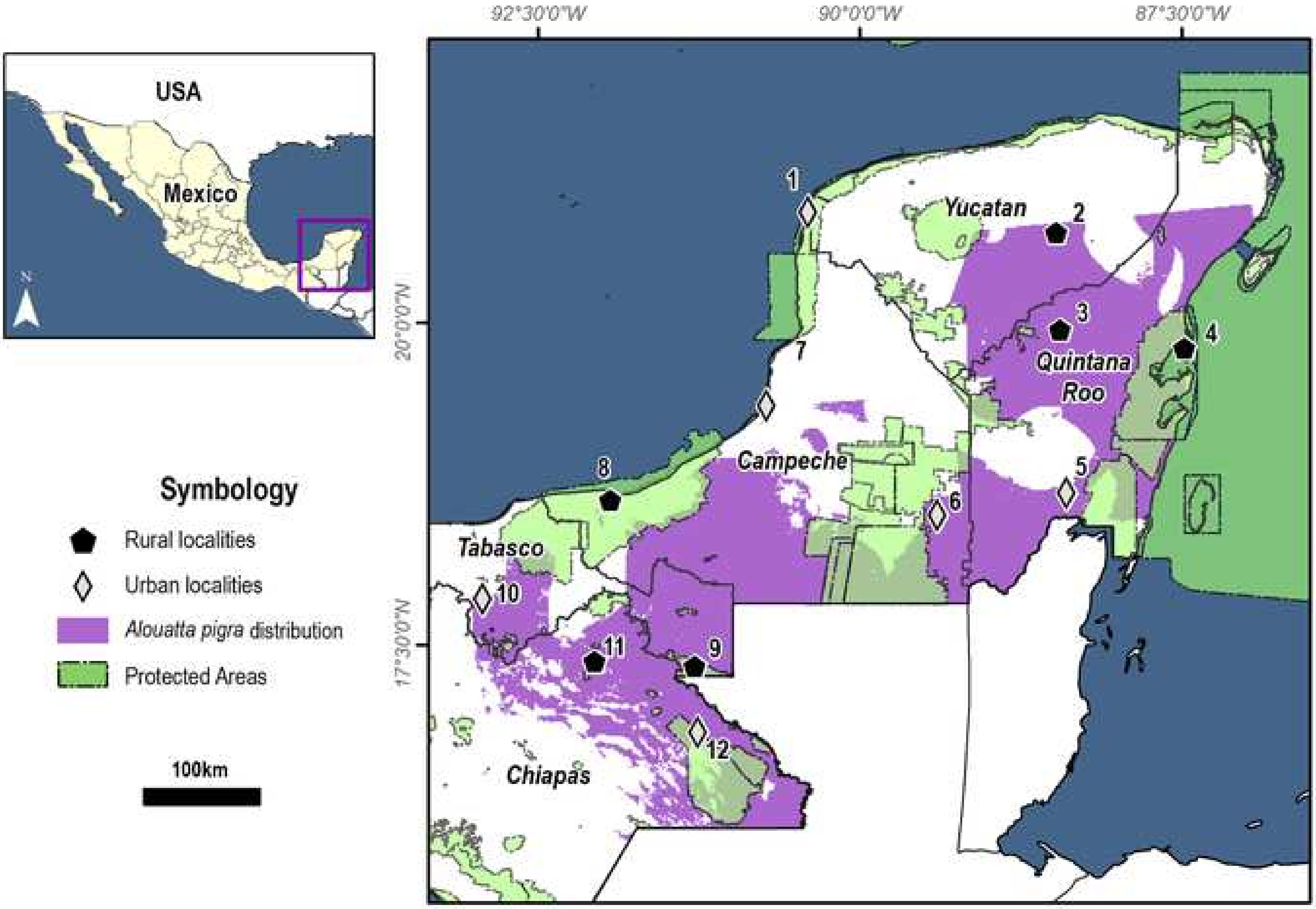

Figure 2

Click here to download Figure Fig 2.tif

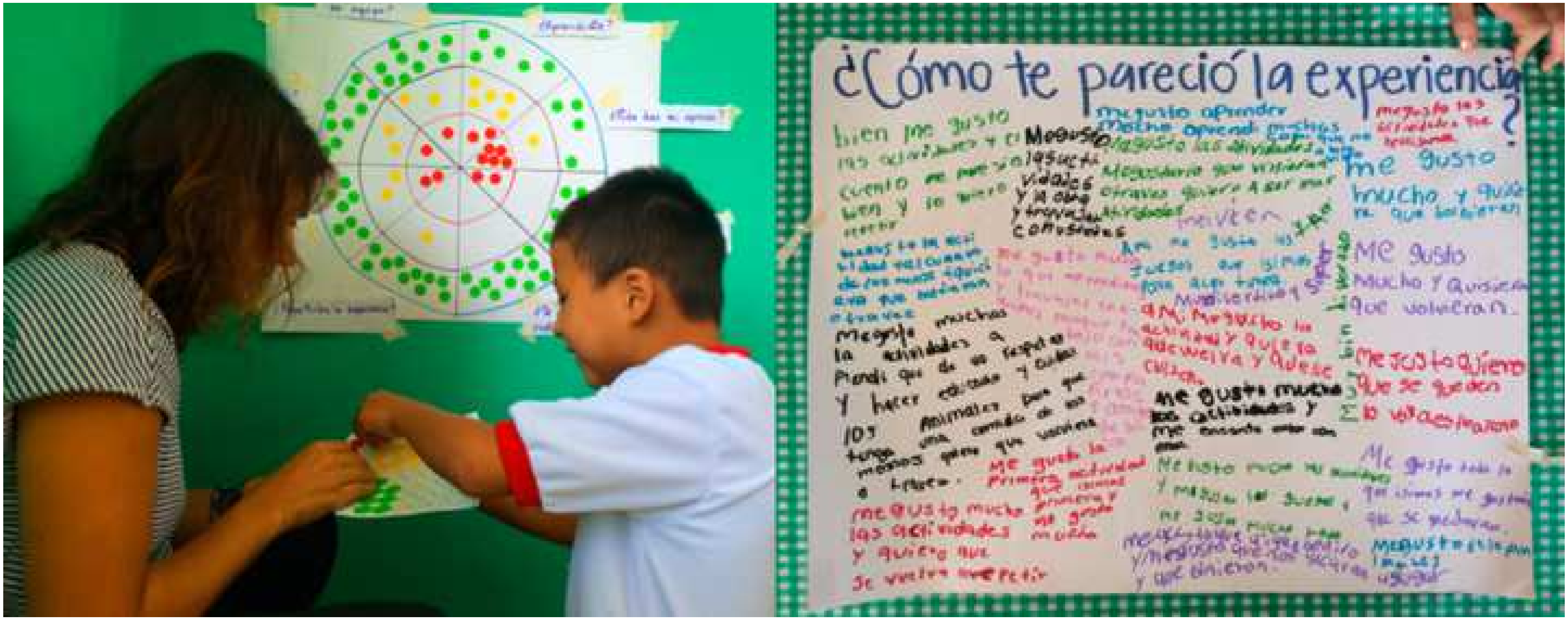

Figure 3

Click here to download Figure Fig 3.tiff

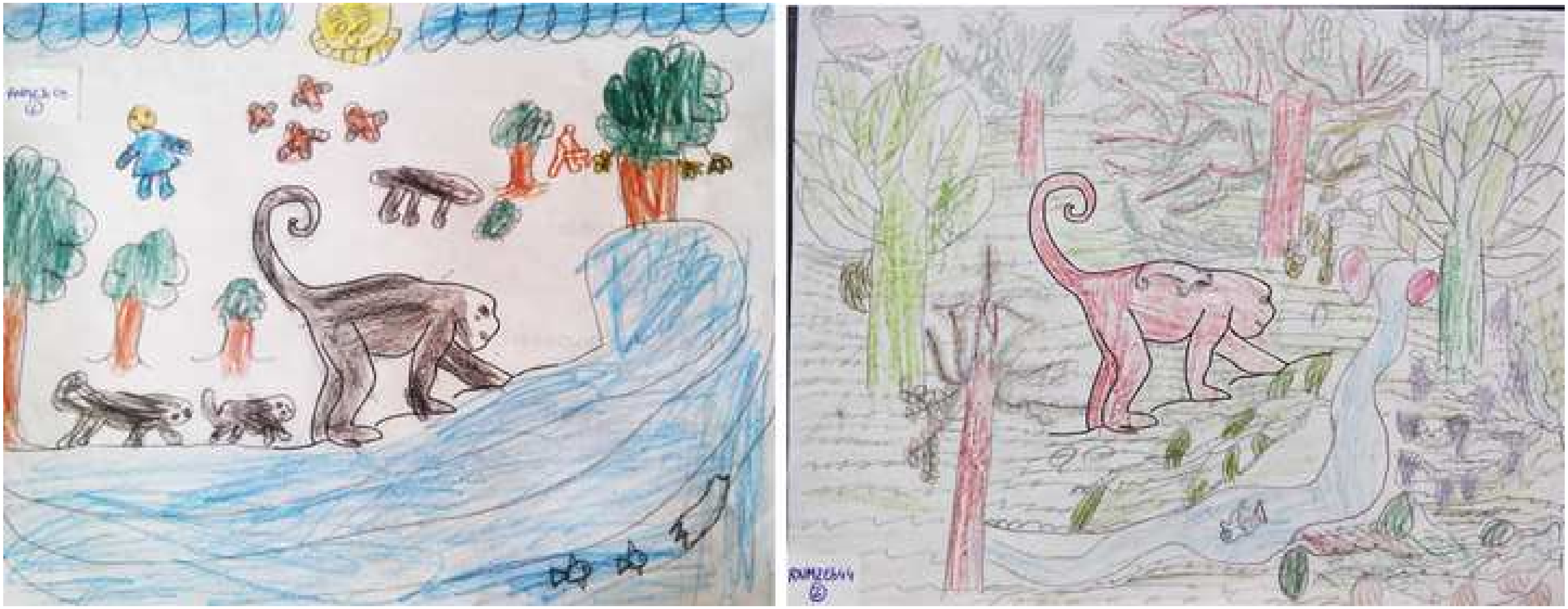

Figure 4

Click here to download Figure Fig 4.tif

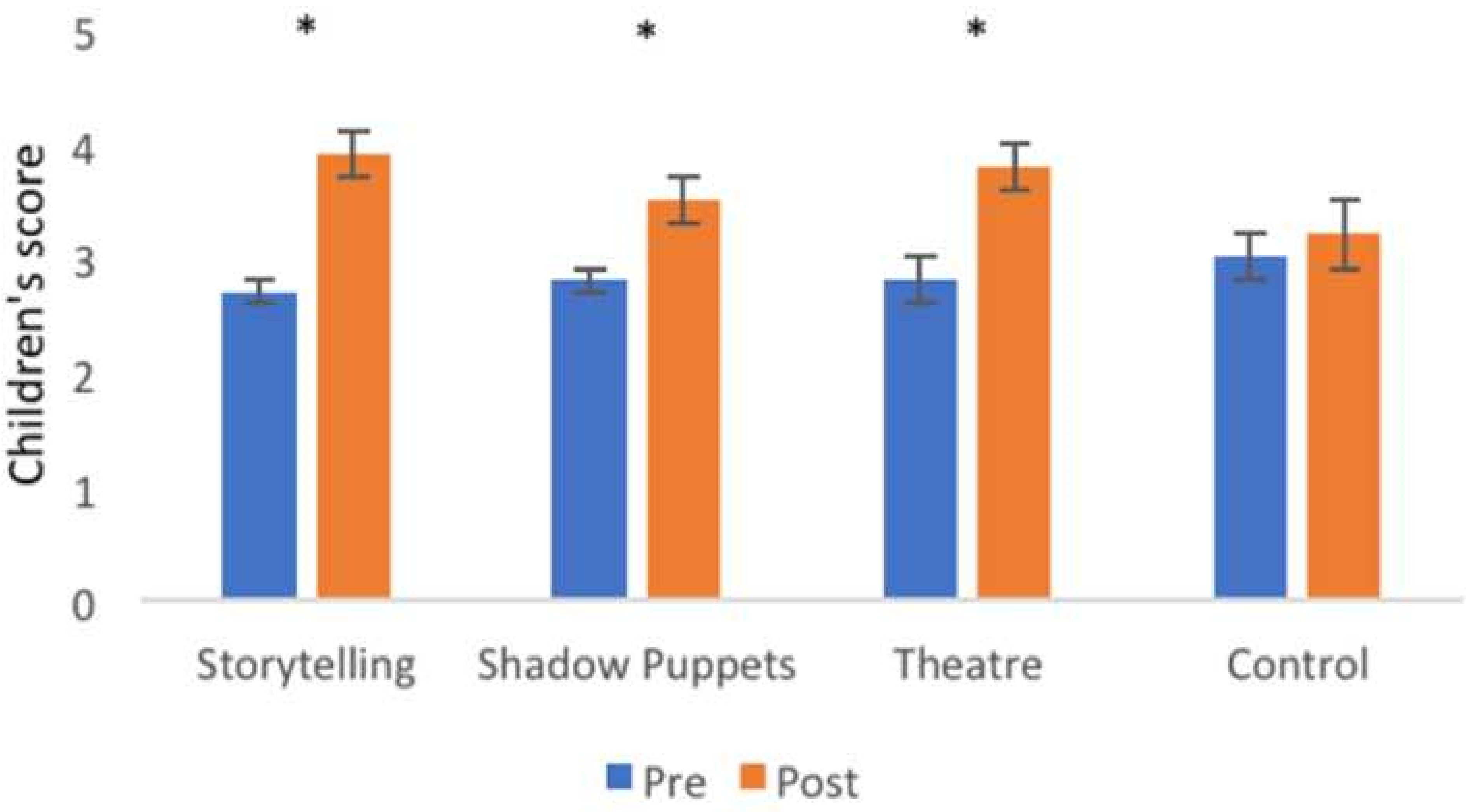

Figure 5

Click here to download Figure Fig 5.tif

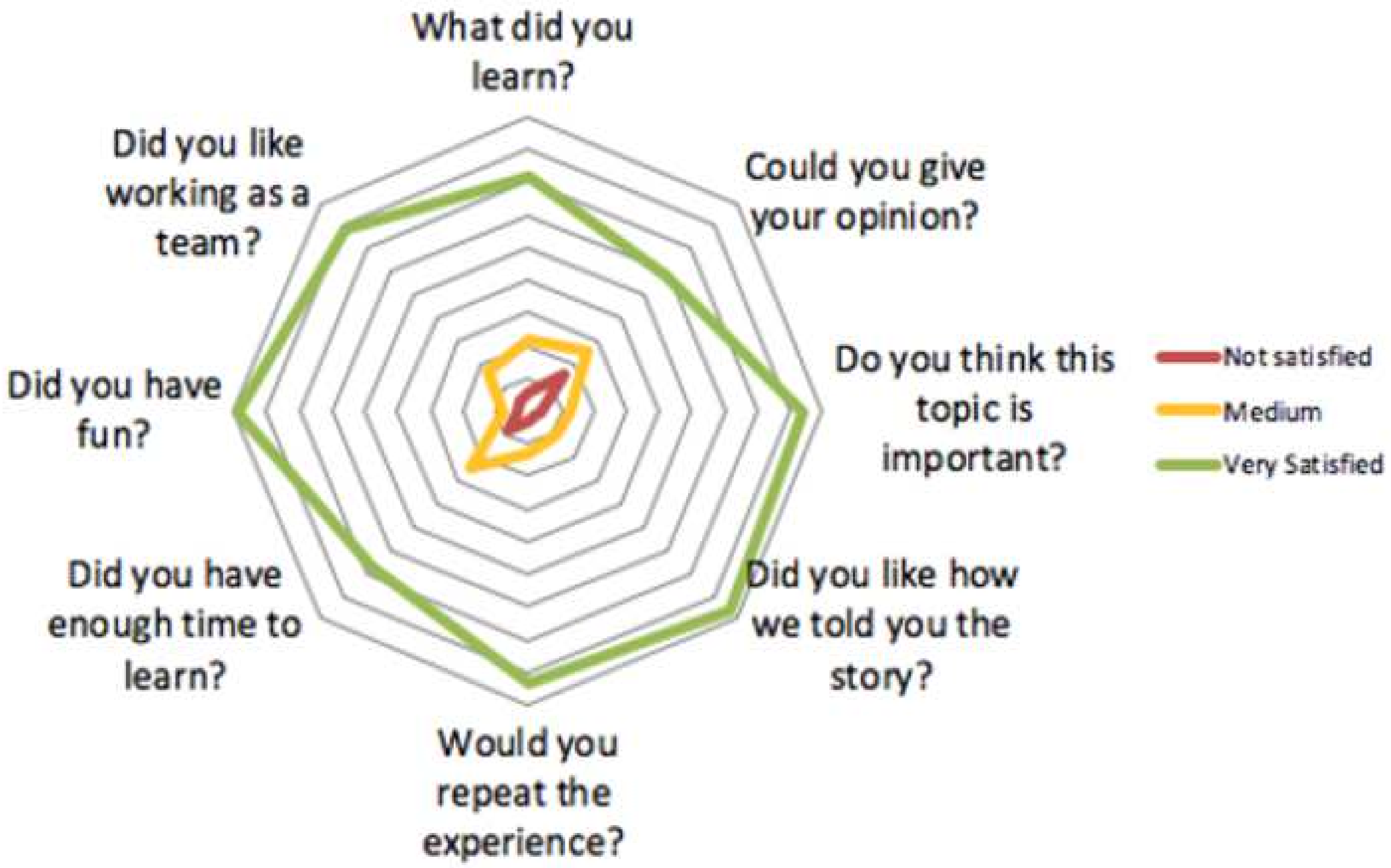

Example of permits obtained

Click here to access/download
Supporting Information
renamed_9391d.jpg
